# Supplementary material for: County medical community, medical insurance package payment, and hierarchical diagnosis and treatment—Empirical analysis of the impact of the pilot project of compact county medical communities in Sichuan Province
Source: PLoS One. 2024 Apr 5;19(4):e0297340. doi: 10.1371/journal.pone.0297340 (PMC10997099; doi:10.1371/journal.pone.0297340)
Supplement: S1 File — (DOCX) [file pone.0297340.s001.docx]

Placebo test (I)

Table 1 Placebo test of the influence of county medical community construction on patient medical diversion

| Explanatory variable | Dependent Variables | | | | |
| --- | --- | --- | --- | --- | --- |
|  | *dtp* | | | *dtr* | |
|  | Medical and health institutions | Public hospitals | Primary medical institutions | Public hospitals | Primary medical institutions |
| *did* | 0.277 | 0.108 | 0.167 | 0.012 | -0.012 |
|  | (0.233) | (0.101) | (0.172) | (0.014) | (0.014) |
| Constant | -5.100 | -2.375 | -2.234 | 0.493 | 0.507 |
|  | (4.939) | (2.203) | (3.673) | (0.317) | (0.317) |
| Annual effect | 390 | 387 | 390 | 390 | 390 |
| Individual effect | 0.900 | 0.949 | 0.881 | 0.945 | 0.945 |
| *R-squared* | yes | yes | yes | yes | yes |
| Sample capacity | yes | yes | yes | yes | yes |

Robust standard errors in parentheses，*** p<0.01, ** p<0.05, * p<0.1

Table 2 Effect of the construction of county medical community on the diagnosis and treatment behavior of medical institutions at all levels

| Explanatory variable | Dependent Variables | | | | | | |
| --- | --- | --- | --- | --- | --- | --- | --- |
|  | *htp* | | | *htr* | | *htsr* | |
|  | Medical and health institutions | Public hospitals | Primary medical institutions | Public hospitals | Primary medical institutions | Hospitals | Public hospitals |
| ygtdid | 0.014 | 0.010 | 0.006** | 0.014 | 0.012 | 0.032 | 0.035* |
|  | (0.007) | (0.005) | (0.003) | (0.012) | (0.008) | (0.017) | (0.021) |
| Constant | -0.309* | -0.064 | -0.167** | 1.281*** | -0.297 | 2.449*** | 2.690*** |
|  | (0.182) | (0.104) | (0.085) | (0.315) | (0.240) | (0.687) | (0.812) |
| Observations | 390 | 387 | 390 | 387 | 390 | 390 | 387 |
| R-squared | 0.921 | 0.955 | 0.914 | 0.964 | 0.970 | 0.706 | 0.661 |
| year FE | yes | yes | yes | yes | yes | yes | yes |
| city FE | yes | yes | yes | yes | yes | yes | yes |

Robust standard errors in parentheses，*** p<0.01, ** p<0.05, * p<0.1

Table 3 Impact of county medical community construction on medical resource allocation Placebo test

| Explanatory variable | Dependent Variables | | | | | | | | |
| --- | --- | --- | --- | --- | --- | --- | --- | --- | --- |
|  | *pher* | | | *phbed* | | | | | |
|  | Hospitals | Health clinics in towns and townships | Community health service centers | Public hospitals | Commun-ity health service centers | Commun-ity health service station | Health clinics in towns and townships | | Outpatient department |
| ygtdid | -21.483 | -9.063 | 3.020 | -16.693 | -1.548 | 2.049** | | -6.339 | -0.584 |
|  | (17.624) | (13.112) | (5.915) | (15.893) | (2.483) | (0.792) | | (14.608) | (0.534) |
| Constant | -726.025** | 675.755 | 106.624 | -34.078 | 26.802 | -0.733 | | -208.728 | 18.360** |
|  | (357.306) | (459.976) | (175.133) | (284.912) | (30.964) | (21.117) | | (184.233) | (8.897) |
| Observations | 392 | 360 | 304 | 392 | 392 | 392 | | 392 | 392 |
| R-squared | 0.965 | 0.900 | 0.851 | 0.928 | 0.729 | 0.834 | | 0.770 | 0.474 |
| year FE | yes | yes | yes | yes | yes | yes | | yes | yes |
| city FE | yes | yes | yes | yes | yes | yes | | yes | yes |

Robust standard errors in parentheses，*** p<0.01, ** p<0.05, * p<0.1

Placebo test (II)

Nuclear density test plots of the explained variables

（1）*dtp* of Medical and health institutions （2）*dtp* of Primary medical institutions

（3）*htp* of Medical and health institutions （4）*htp* of Public hospitals

（5）*htp* of Primary medical institutions （6）*htsr* of Hospitals

（7）*htsr* of Public hospitals （8）*phbed* of Community health service station
