# Supplementary material for: County medical community, medical insurance package payment, and hierarchical diagnosis and treatment—Empirical analysis of the impact of the pilot project of compact county medical communities in Sichuan Province
Source: PLoS One. 2024 Apr 5;19(4):e0297340. doi: 10.1371/journal.pone.0297340 (PMC10997099; doi:10.1371/journal.pone.0297340)
Supplement: S3 File — (DOCX) [file pone.0297340.s003.docx]

*Descriptive Statistical Analysis

su y0 h0 tf0066100 tf0046103 tf0045103 tf0071100 tf0046104 tf0045104 tf0070102 tf0071102 tc0015100 tc0016101 tc0017100 td0022102 td0022109 td0022112 if ifygt==0

su y0 h0 tf0066100 tf0046103 tf0045103 tf0071100 tf0046104 tf0045104 tf0070102 tf0071102 tc0015100 tc0016101 tc0017100 td0022102 td0022109 td0022112 if ifygt==1

su agingdegree1 sexrate urpopustr covid_19 lngdp if ifygt==0

su agingdegree1 sexrate urpopustr covid_19 lngdp if ifygt==1

*Diversion Effect

*PSM

clear

use "C:\S4 File. Data sample.dta"

gen ygtt=0

replace ygtt=1 if year>=2019

g ygtdid=ygtt*ifygt

set seed 0001

gen tmp = runiform() //生成随机数

sort tmp

psmatch2 ifygt agingdegree1 sexrate urpopustr covid_19 lngdp tc0015100,logit ate neighbor(1) common caliper(.05) ties

pstest agingdegree1 sexrate urpopustr covid_19 lngdp tc0015100,both graph

drop if _weight ==.

*DID-Number of consultations per capita(dtp)

reghdfe y0 ygtdid agingdegree1 urpopustr lngdp, absorb(id year) vce(r) //人均诊疗人次 显著正向

est sto s1

reghdfe tf0066100 ygtdid agingdegree1 urpopustr lngdp, absorb(id year) vce(r) //公立医院诊疗人次

est sto s2

reghdfe tf0045103 ygtdid agingdegree1 urpopustr lngdp, absorb(id year) vce(r) //基层医疗机构诊疗人次 显著正向

est sto s3

esttab s1 s2 s3 ,ar2(%8.4f)se(%8.4f) star(* 0.1 ** 0.05 *** 0.01) aic bic mtitles

outreg2[s1 s2 s3] using Diversion Effect(dtp).doc, bdec(3) sdec(3) addtext(year FE,yes,city FE,yes)

*DID-The proportion of consultations in different levels of medical institutions(dtr)

reghdfe y3 ygtdid agingdegree1 urpopustr lngdp, absorb(id year) vce(r) //公立医院诊疗人次占比

est sto s1

reghdfe y1 ygtdid agingdegree1 urpopustr lngdp, absorb(id year) vce(r) //基层医疗机构诊疗人次占比

est sto s2

esttab s1 s2,ar2(%8.4f)se(%8.4f) star(* 0.1 ** 0.05 *** 0.01) aic bic mtitles

outreg2[s1 s2 ] using Diversion Effect(dtr).doc, bdec(3) sdec(3) addtext(year FE,yes,city FE,yes)

*Parallel trend test

gen policy = year - 2019

tab policy

//首先生成年份虚拟变量与实验组虚拟变量的交互项

forvalues i = 3(-1)1{

gen pre_`i' = (policy == -`i' & ifygt == 1)

}

gen current = (policy == 0 & ifygt == 1)

forvalues j = 1(1)2{

gen post_`j' = (policy == `j' & ifygt == 1)

}

drop pre_1 //将政策前第一期作为基准组，很重要！！！

global x1 "agingdegree1 urpopustr lngdp "

reghdfe y0 pre* current post* $x1, absorb(id year) vce(r)

est sto s1

reghdfe tf0066100 pre* current post* $x1, absorb(id year) vce(r)

est sto s2

reghdfe tf0045103 pre* current post* $x1, absorb(id year) vce(r)

est sto s3

esttab s1 s2 s3,ar2(%8.4f)se(%8.4f) star(* 0.1 ** 0.05 *** 0.01) aic bic mtitles

outreg2[s1 s2 s3] using P-dtp.doc, bdec(3) sdec(3) addtext(year FE,yes,city FE,yes)

*绘图

coefplot, baselevels ///

keep(pre* current post*) ///

vertical ///转置图形

coeflabels(pre5=-5 pre4=-4 pre3=-3 pre2=-2 pre1=-1 ///

current=0 post1=1 post2=2 post3=3 ) ///

yline(0,lwidth(vthin) lpattern(solid) lcolor(teal)) ///

xline(3,lwidth(vthin) lpattern(dash) lcolor(teal)) ///

ylabel(-0.2(0.2)0.4,labsize(*0.85) angle(0)) xlabel(,labsize(*0.85)) ///

ytitle("政策动态效应", size(small)) ///加入Y轴标题,大小small

xtitle("政策时点", size(small)) ///加入X轴标题，大小small

msymbol(O) msize(small) mcolor(gs1) ///plot样式

addplot(line @b @at,lcolor(gs1) lwidth(medthick)) ///增加点之间的连线

ciopts(lpattern(solid) recast(rcap) msize(medium)) ///置信区间样式

graphregion(color(white)) //白底

drop policy pre* current post*

*Diagnosis and treatment effect

*PSM

clear

use "C:\S4 File. Data sample.dta"

gen ygtt=0

replace ygtt=1 if year>=2019

g ygtdid=ygtt*ifygt

set seed 0001

gen tmp = runiform() //生成随机数

sort tmp

psmatch2 ifygt agingdegree1 sexrate urpopustr covid_19 lngdp tc0015100,logit ate neighbor(1) common caliper(.05) ties

pstest agingdegree1 sexrate urpopustr covid_19 lngdp tc0015100,both graph

drop if _weight ==.

*Diagnosis and treatment effect-Number of hospitalizations per capita (htp)

reghdfe h0 ygtdid agingdegree1 urpopustr lngdp, absorb(id year) vce(r) //人均入院人次 显著正向

est sto s1

reghdfe tf0071100 ygtdid agingdegree1 urpopustr lngdp, absorb(id year) vce(r) //公立入院人次 显著正向

est sto s2

reghdfe tf0045104 ygtdid agingdegree1 urpopustr lngdp, absorb(id year) vce(r) //基层医疗机构入院人次 did显著正向

est sto s3

esttab s1 s2 s3 ,ar2(%8.4f)se(%8.4f) star(* 0.1 ** 0.05 *** 0.01) aic bic mtitles

outreg2[s1 s2 s3 ] using 门诊统筹诊疗效应a-1.doc, bdec(3) sdec(3) addtext(year FE,yes,city FE,yes)

*Diagnosis and treatment effect-The proportion of inpatients in different levels of medical institutions(htr)

reghdfe h3 ygtdid agingdegree1 urpopustr lngdp, absorb(id year) vce(r) //公立入院人次占比

est sto s1

reghdfe h1 ygtdid agingdegree1 urpopustr lngdp, absorb(id year) vce(r) //基层医疗机构入院人次占比 did显著正向

est sto s2

esttab s1 s2 ,ar2(%8.4f)se(%8.4f) star(* 0.1 ** 0.05 *** 0.01) aic bic mtitles

outreg2[s1 s2 ] using 门诊统筹诊疗效应f.doc, bdec(3) sdec(3) addtext(year FE,yes,city FE,yes)

*Diagnosis and treatment effect-Surgery rate in hospitalized patients(htsr)

reghdfe tf0070102 ygtdid agingdegree1 urpopustr lngdp, absorb(id year) vce(r) //医院住院病人手术率 显著正向

est sto s1

reghdfe tf0071102 ygtdid agingdegree1 urpopustr lngdp, absorb(id year) vce(r) //公立住院病人手术率 显著正向

est sto s2

esttab s1 s2 ,ar2(%8.4f)se(%8.4f) star(* 0.1 ** 0.05 *** 0.01) aic bic mtitles

outreg2[s1 s2 ] using 门诊统筹诊疗效应c.doc, bdec(3) sdec(3) addtext(year FE,yes,city FE,yes)

*Parallel trend test

gen policy = year - 2019

tab policy

//首先生成年份虚拟变量与实验组虚拟变量的交互项

forvalues i = 3(-1)1{

gen pre_`i' = (policy == -`i' & ifygt == 1)

}

gen current = (policy == 0 & ifygt == 1)

forvalues j = 1(1)2{

gen post_`j' = (policy == `j' & ifygt == 1)

}

drop pre_1 //将政策前第一期作为基准组，很重要！！！

global x1 "agingdegree1 urpopustr lngdp "

reghdfe h0 pre* current post* $x1, absorb(id year) vce(r) //显著正向

est sto s1

reghdfe tf0071100 pre* current post* $x1, absorb(id year) vce(r) //显著正向

est sto s2

reghdfe tf0045104 pre* current post* $x1, absorb(id year) vce(r)

est sto s3

esttab s1 s2 s3 ,ar2(%8.4f)se(%8.4f) star(* 0.1 ** 0.05 *** 0.01) aic bic mtitles

outreg2[s1 s2 s3 ] using p-htp.doc, bdec(3) sdec(3) addtext(year FE,yes,city FE,yes)

reghdfe h3 pre* current post* $x1, absorb(id year) vce(r) //显著正向

est sto s1

reghdfe h1 pre* current post* $x1, absorb(id year) vce(r) //显著正向

est sto s2

esttab s1 s2,ar2(%8.4f)se(%8.4f) star(* 0.1 ** 0.05 *** 0.01) aic bic mtitles

outreg2[s1 s2] using p-htr.doc, bdec(3) sdec(3) addtext(year FE,yes,city FE,yes)

reghdfe tf0070102 pre* current post* $x1, absorb(id year) vce(r) //显著正向

est sto s1

reghdfe tf0071102 pre* current post* $x1, absorb(id year) vce(r) //显著正向

est sto s2

esttab s1 s2 ,ar2(%8.4f)se(%8.4f) star(* 0.1 ** 0.05 *** 0.01) aic bic mtitles

outreg2[s1 s2 ] using p-htsr.doc, bdec(3) sdec(3) addtext(year FE,yes,city FE,yes)

*Resource allocation effect

*Health workers per thousand people(pher)

clear

use "C:\S4 File. Data sample.dta"

set seed 0001

gen tmp = runiform() //生成随机数

sort tmp

psmatch2 ifygt agingdegree1 sexrate urpopustr covid_19 lngdp ,outcome(tc0015100) logit ate neighbor(1) common caliper(.05) ties

pstest agingdegree1 sexrate urpopustr covid_19 lngdp ,both graph

drop if _weight ==.

gen ygtt=0

replace ygtt=1 if year>=2019

g ygtdid=ygtt*ifygt

reghdfe tc0015100 ygtdid agingdegree1 lngdp, absorb(id year) vce(r) //医院卫生技术人员

est sto s1

esttab s1 ,ar2(%8.4f)se(%8.4f) star(* 0.1 ** 0.05 *** 0.01) aic bic mtitles

outreg2[s1 ] using phbed1.doc, bdec(3) sdec(3) addtext(year FE,yes,city FE,yes)

*Parallel trend test

gen policy = year - 2019

tab policy

//首先生成年份虚拟变量与实验组虚拟变量的交互项

forvalues i = 3(-1)1{

gen pre_`i' = (policy == -`i' & ifygt == 1)

}

gen current = (policy == 0 & ifygt == 1)

forvalues j = 1(1)2{

gen post_`j' = (policy == `j' & ifygt == 1)

}

drop pre_1 //将政策前第一期作为基准组，很重要！！！

global x2 "agingdegree1 lngdp "

reghdfe tc0015100 pre* current post* $x2 , absorb(id year) vce(r)

est sto s1

esttab s1 ,ar2(%8.4f)se(%8.4f) star(* 0.1 ** 0.05 *** 0.01) aic bic mtitles

outreg2[s1 ] using p-phbed1.doc, bdec(3) sdec(3) addtext(year FE,yes,city FE,yes)

clear

use "C:\S4 File. Data sample.dta"

set seed 0001

gen tmp = runiform() //生成随机数

sort tmp

psmatch2 ifygt agingdegree1 sexrate urpopustr covid_19 lngdp ,outcome(tc0016101) logit ate neighbor(1) common caliper(.05) ties

pstest agingdegree1 sexrate urpopustr covid_19 lngdp ,both graph

drop if _weight ==.

gen ygtt=0

replace ygtt=1 if year>=2019

g ygtdid=ygtt*ifygt

reghdfe tc0016101 ygtdid agingdegree1 sexrate urpopustr lngdp, absorb(id year) vce(r) //乡镇卫生院卫生技术人员

est sto s1

esttab s1 ,ar2(%8.4f)se(%8.4f) star(* 0.1 ** 0.05 *** 0.01) aic bic mtitles

outreg2[s1] using phbed2.doc, bdec(3) sdec(3) addtext(year FE,yes,city FE,yes)

*Parallel trend test

gen policy = year - 2019

tab policy

//首先生成年份虚拟变量与实验组虚拟变量的交互项

forvalues i = 3(-1)1{

gen pre_`i' = (policy == -`i' & ifygt == 1)

}

gen current = (policy == 0 & ifygt == 1)

forvalues j = 1(1)2{

gen post_`j' = (policy == `j' & ifygt == 1)

}

drop pre_1 //将政策前第一期作为基准组，很重要！！！

global x2 "agingdegree1 lngdp "

reghdfe tc0016101 pre* current post* $x2 , absorb(id year) vce(r)

est sto s1

esttab s1 ,ar2(%8.4f)se(%8.4f) star(* 0.1 ** 0.05 *** 0.01) aic bic mtitles

outreg2[s1 ] using p-phbed2.doc, bdec(3) sdec(3) addtext(year FE,yes,city FE,yes)

clear

use "C:\S4 File. Data sample.dta"

set seed 0001

gen tmp = runiform() //生成随机数

sort tmp

psmatch2 ifygt agingdegree1 sexrate urpopustr covid_19 lngdp ,outcome(tc0017101) logit ate neighbor(1) common caliper(.05) ties

pstest agingdegree1 sexrate urpopustr covid_19 lngdp ,both graph

drop if _weight ==.

gen ygtt=0

replace ygtt=1 if year>=2019

g ygtdid=ygtt*ifygt

reghdfe tc0017100 ygtdid sexrate lngdp, absorb(id year) vce(r) //社区卫生服务中心卫生技术人员

est sto s1

esttab s1 ,ar2(%8.4f)se(%8.4f) star(* 0.1 ** 0.05 *** 0.01) aic bic mtitles

outreg2[s1] using phbed3.doc, bdec(3) sdec(3) addtext(year FE,yes,city FE,yes)

*Parallel trend test

gen policy = year - 2019

tab policy

//首先生成年份虚拟变量与实验组虚拟变量的交互项

forvalues i = 3(-1)1{

gen pre_`i' = (policy == -`i' & ifygt == 1)

}

gen current = (policy == 0 & ifygt == 1)

forvalues j = 1(1)2{

gen post_`j' = (policy == `j' & ifygt == 1)

}

drop pre_1 //将政策前第一期作为基准组，很重要！！！

global x2 "agingdegree1 lngdp "

reghdfe tc0017101 pre* current post* $x2 , absorb(id year) vce(r)

est sto s1

esttab s1 ,ar2(%8.4f)se(%8.4f) star(* 0.1 ** 0.05 *** 0.01) aic bic mtitles

outreg2[s1 ] using p-phbed3.doc, bdec(3) sdec(3) addtext(year FE,yes,city FE,yes)

*Resource allocation effect

*PSM

clear

use "C:\S4 File. Data sample.dta"

gen ygtt=0

replace ygtt=1 if year>=2019

g ygtdid=ygtt*ifygt

set seed 0001

gen tmp = runiform() //生成随机数

sort tmp

psmatch2 ifygt agingdegree1 sexrate urpopustr covid_19 lngdp , logit ate neighbor(1) common caliper(.05) ties

pstest agingdegree1 sexrate urpopustr covid_19 lngdp ,both graph

drop if _weight ==.

*Number of beds per thousand people(phbed)

reghdfe td0022101 ygtdid agingdegree1 lngdp , absorb(id year) vce(r) //公立医院小计

est sto s0

reghdfe td0022108 ygtdid agingdegree1 lngdp , absorb(id year) vce(r) //社区卫生服务中心

est sto s1

reghdfe td0022109 ygtdid agingdegree1 lngdp , absorb(id year) vce(r) //社区卫生服务站 显著正向

est sto s2

reghdfe td0022111 ygtdid agingdegree1 lngdp , absorb(id year) vce(r) //乡镇卫生院

est sto s3

reghdfe td0022112 ygtdid agingdegree1 lngdp , absorb(id year) vce(r) //门诊部 显著负向

est sto s4

esttab s0 s1 s2 s3 s4 ,ar2(%8.4f)se(%8.4f) star(* 0.1 ** 0.05 *** 0.01) aic bic mtitles

outreg2[s0 s1 s2 s3 s4] using pher.doc, bdec(3) sdec(3) addtext(year FE,yes,city FE,yes)

*Parallel trend test

gen policy = year - 2019

tab policy

//首先生成年份虚拟变量与实验组虚拟变量的交互项

forvalues i = 3(-1)1{

gen pre_`i' = (policy == -`i' & ifygt == 1)

}

gen current = (policy == 0 & ifygt == 1)

forvalues j = 1(1)2{

gen post_`j' = (policy == `j' & ifygt == 1)

}

drop pre_1 //将政策前第一期作为基准组，很重要！！！

global x2 "agingdegree1 lngdp "

reghdfe td0022101 pre* current post* $x2 , absorb(id year) vce(r)

est sto s1

reghdfe td0022108 pre* current post* $x2 , absorb(id year) vce(r)

est sto s2

reghdfe td0022109 pre* current post* $x2 , absorb(id year) vce(r) //显著正向

est sto s3

reghdfe td0022111 pre* current post* $x2 , absorb(id year) vce(r)

est sto s4

reghdfe td0022112 pre* current post* $x2 , absorb(id year) vce(r)

est sto s5

esttab s1 s2 s3 s4 s5,ar2(%8.4f)se(%8.4f) star(* 0.1 ** 0.05 *** 0.01) aic bic mtitles

outreg2[s1 s2 s3 s4 s5] using 资源配置平行趋势检验b.doc, bdec(3) sdec(3) addtext(year FE,yes,city FE,yes)

*Placebo test（1）

*PSM

clear

use "C:\S4 File. Data sample.dta"

gen ygtt=0

replace ygtt=1 if year>=2017

g ygtdid=ygtt*ifygt

set seed 0001

gen tmp = runiform() //生成随机数

sort tmp

psmatch2 ifygt agingdegree1 sexrate urpopustr covid_19 lngdp tc0015100,logit ate neighbor(1) common caliper(.05) ties

pstest agingdegree1 sexrate urpopustr covid_19 lngdp tc0015100,both graph

drop if _weight ==.

*DID

*Diversion effect

*Number of consultations per capita(dtp)

reghdfe y0 ygtdid agingdegree1 urpopustr lngdp, absorb(id year) vce(r) //人均诊疗人次

est sto s0

reghdfe tf0065100 ygtdid agingdegree1 urpopustr lngdp, absorb(id year) vce(r) //医院诊疗人次

est sto s1

reghdfe tf0045103 ygtdid agingdegree1 urpopustr lngdp, absorb(id year) vce(r) //基层医疗机构诊疗人次

est sto s2

esttab s0 s1 s2 ,ar2(%8.4f)se(%8.4f) star(* 0.1 ** 0.05 *** 0.01) aic bic mtitles

outreg2[s0 s1 s2 ] using Placebo test（1）(dtp).doc, bdec(3) sdec(3) addtext(year FE,yes,city FE,yes)

*The proportion of diagnosis and treatment(dtr)

reghdfe y2 ygtdid agingdegree1 urpopustr lngdp, absorb(id year) vce(r) //公立诊疗人次占比

est sto s1

reghdfe y1 ygtdid agingdegree1 urpopustr lngdp, absorb(id year) vce(r) //基层医疗机构诊疗人次占比

est sto s2

esttab s1 s2 ,ar2(%8.4f)se(%8.4f) star(* 0.1 ** 0.05 *** 0.01) aic bic mtitles

outreg2[s1 s2 ] using Placebo test（1）(dtr).doc, bdec(3) sdec(3) addtext(year FE,yes,city FE,yes)

*Diagnosis and treatment effect

*Number of hospitalizations per capita(htp)

reghdfe h0 ygtdid agingdegree1 urpopustr lngdp, absorb(id year) vce(r) //人均入院人次

est sto s0

reghdfe tf0071100 ygtdid agingdegree1 urpopustr lngdp, absorb(id year) vce(r) //公立入院人次

est sto s1

reghdfe tf0045104 ygtdid agingdegree1 urpopustr lngdp, absorb(id year) vce(r) //基层医疗机构入院人次 显著正向

est sto s2

esttab s0 s1 s2 ,ar2(%8.4f)se(%8.4f) star(* 0.1 ** 0.05 *** 0.01) aic bic mtitles

outreg2[s0 s1 s2 ] using Placebo test（1）(htp).doc, bdec(3) sdec(3) addtext(year FE,yes,city FE,yes)

*The proportion of inpatients(htr)

reghdfe h3 ygtdid agingdegree1 urpopustr lngdp, absorb(id year) vce(r) //公立入院人次占比

est sto s1

reghdfe h1 ygtdid agingdegree1 urpopustr lngdp, absorb(id year) vce(r) //基层医疗机构入院人次占比

est sto s2

esttab s1 s2 ,ar2(%8.4f)se(%8.4f) star(* 0.1 ** 0.05 *** 0.01) aic bic mtitles

outreg2[s1 s2 ] using Placebo test（1）(htr).doc, bdec(3) sdec(3) addtext(year FE,yes,city FE,yes)

*Surgery rate in hospitalized patients(htsr)

reghdfe tf0070102 ygtdid agingdegree1 urpopustr lngdp, absorb(id year) vce(r) //医院住院病人手术率 显著正向

est sto s1

reghdfe tf0071102 ygtdid agingdegree1 urpopustr lngdp, absorb(id year) vce(r) //公立住院病人手术率 显著正向

est sto s2

esttab s1 s2 ,ar2(%8.4f)se(%8.4f) star(* 0.1 ** 0.05 *** 0.01) aic bic mtitles

outreg2[s1 s2 ] using Placebo test（1）(htsr).doc, bdec(3) sdec(3) addtext(year FE,yes,city FE,yes)

clear

use "C:\S4 File. Data sample.dta"

gen ygtt=0

replace ygtt=1 if year>=2017

g ygtdid=ygtt*ifygt

set seed 0001

gen tmp = runiform() //生成随机数

sort tmp

psmatch2 ifygt agingdegree1 sexrate urpopustr covid_19 lngdp , logit ate neighbor(1) common caliper(.05) ties

pstest agingdegree1 sexrate urpopustr covid_19 lngdp ,both graph

drop if _weight ==.

*Health workers per thousand people(pher)

clear

use "C:\S4 File. Data sample.dta"

set seed 0001

gen tmp = runiform() //生成随机数

sort tmp

psmatch2 ifygt agingdegree1 sexrate urpopustr covid_19 lngdp ,outcome(tc0015100) logit ate neighbor(1) common caliper(.05) ties

pstest agingdegree1 sexrate urpopustr covid_19 lngdp ,both graph

drop if _weight ==.

gen ygtt=0

replace ygtt=1 if year>=2017

g ygtdid=ygtt*ifygt

reghdfe tc0015100 ygtdid agingdegree1 lngdp, absorb(id year) vce(r) //医院卫生技术人员

est sto s1

esttab s1 ,ar2(%8.4f)se(%8.4f) star(* 0.1 ** 0.05 *** 0.01) aic bic mtitles

outreg2[s1 ] using Placebo test（1）(pher)-1.doc, bdec(3) sdec(3) addtext(year FE,yes,city FE,yes)

clear

use "C:\S4 File. Data sample.dta"

set seed 0001

gen tmp = runiform() //生成随机数

sort tmp

psmatch2 ifygt agingdegree1 sexrate urpopustr covid_19 lngdp ,outcome(tc0016101) logit ate neighbor(1) common caliper(.05) ties

pstest agingdegree1 sexrate urpopustr covid_19 lngdp ,both graph

drop if _weight ==.

gen ygtt=0

replace ygtt=1 if year>=2017

g ygtdid=ygtt*ifygt

reghdfe tc0016101 ygtdid agingdegree1 sexrate urpopustr lngdp, absorb(id year) vce(r) //乡镇卫生院卫生技术人员

est sto s1

esttab s1 ,ar2(%8.4f)se(%8.4f) star(* 0.1 ** 0.05 *** 0.01) aic bic mtitles

outreg2[s1] using Placebo test（1）(pher)-2.doc, bdec(3) sdec(3) addtext(year FE,yes,city FE,yes)

clear

use "C:\S4 File. Data sample.dta"

set seed 0001

gen tmp = runiform() //生成随机数

sort tmp

psmatch2 ifygt agingdegree1 sexrate urpopustr covid_19 lngdp ,outcome(tc0017101) logit ate neighbor(1) common caliper(.05) ties

pstest agingdegree1 sexrate urpopustr covid_19 lngdp ,both graph

drop if _weight ==.

gen ygtt=0

replace ygtt=1 if year>=2017

g ygtdid=ygtt*ifygt

reghdfe tc0017100 ygtdid sexrate lngdp, absorb(id year) vce(r) //社区卫生服务中心卫生技术人员

est sto s1

esttab s1 ,ar2(%8.4f)se(%8.4f) star(* 0.1 ** 0.05 *** 0.01) aic bic mtitles

outreg2[s1] using Placebo test（1）(pher)-3.doc, bdec(3) sdec(3) addtext(year FE,yes,city FE,yes)

clear

use "C:\S4 File. Data sample.dta"

gen ygtt=0

replace ygtt=1 if year>=2019

g ygtdid=ygtt*ifygt

set seed 0001

gen tmp = runiform() //生成随机数

sort tmp

psmatch2 ifygt agingdegree1 sexrate urpopustr covid_19 lngdp ,outcome(lne0027100) logit ate neighbor(1) common caliper(.05) ties

pstest agingdegree1 sexrate urpopustr covid_19 lngdp ,both graph

drop if _weight ==.

*Number of beds per thousand people(phbed)

reghdfe td0022102 ygtdid agingdegree1 lngdp , absorb(id year) vce(r) //综合医院

est sto s1

reghdfe td0022108 ygtdid agingdegree1 lngdp , absorb(id year) vce(r) //社区卫生服务中心

est sto s2

reghdfe td0022109 ygtdid agingdegree1 lngdp , absorb(id year) vce(r) //社区卫生服务站 显著正向

est sto s3

reghdfe td0022111 ygtdid agingdegree1 lngdp , absorb(id year) vce(r) //乡镇卫生院

est sto s4

reghdfe td0022112 ygtdid agingdegree1 lngdp , absorb(id year) vce(r) //门诊部 显著负向

est sto s5

esttab s1 s2 s3 s4 s5,ar2(%8.4f)se(%8.4f) star(* 0.1 ** 0.05 *** 0.01) aic bic mtitles

outreg2[s1 s2 s3 s4 s5] using Placebo test（1）(phbed).doc, bdec(3) sdec(3) addtext(year FE,yes,city FE,yes)

*Placebo test（2）

*Number of consultations per capita

clear

use "C:\S4 File. Data sample.dta"

set seed 0001

gen tmp = runiform() //生成随机数

sort tmp

psmatch2 ifygt agingdegree1 sexrate urpopustr covid_19 lngdp tc0015100,logit ate neighbor(1) common caliper(.05) ties

pstest agingdegree1 sexrate urpopustr covid_19 lngdp tc0015100,both graph

drop if _weight ==.

reghdfe y0 ygtdid agingdegree1 urpopustr lngdp, absorb(id year) vce(r) //人均诊疗人次 显著正向

duplicates report id year //检查个体-年份是否能唯一识别

duplicates example id year //举例子看哪里数据重复

duplicates drop id year,force //强制删除重复数据

tsset id year //再次设置面板数据

xtdes //查看数据

**生成储存每次循环估计系数、标准误和p值的矩阵

set matsize 999

mat b =J(500,1,0) //系数矩阵

mat se = J(500,1,0) //标准误矩阵

mat p = J(500,1,0) //p值矩阵

**循环500次抽取随机样本并回归

forvalues i = 1/500{

use C:\S4 File. Data sample.dta, clear

**设置面板数据

***由于该数据集中有重复数据，先删去重复数据

duplicates report id year //检查个体-年份是否能唯一识别

duplicates example id year //举例子看哪里数据重复

duplicates drop id year,force //强制删除重复数据

xtset id year //设置面板数据

keep if year ==2015 //保留一期数据

sample 37, count //随机抽取326家快餐店（处理组样本容量为326，可从平衡性分析结果看出）

keep id //得到抽取样本id编号

save match_id.dta, replace //另存id数据

merge 1:m id using "C:\S4 File. Data sample.dta" //与原数据匹配

drop ifygt ygtdid //删掉原本的处理组虚拟变量

gen ifygt = (_merge == 3) //将所抽取样本赋值为1，其余为0，得到伪处理虚拟变量

gen ygtdid =ifygt*ygtt

reghdfe y0 ygtdid agingdegree1 urpopustr lngdp, absorb(id year) vce(r)

* 将回归结果赋值到对应矩阵的对应位置

mat b[`i',1] = _b[ygtdid]

mat se[`i',1] = _se[ygtdid]

* 计算P值并赋值于矩阵

mat p[`i',1] = 2*ttail(e(df_r), abs(_b[ygtdid]/_se[ygtdid]))

}

* 矩阵转化为向量

svmat b, names(coef)

svmat se, names(se)

svmat p, names(pvalue)

* 删除空值并添加标签

drop if pvalue1 == .

label var pvalue1 p值

label var coef1 估计系数

keep coef1 se1 pvalue1

save placebo.dta,replace

*绘图

use placebo.dta,clear

twoway (kdensity coef1) (scatter pvalue1 coef1, msymbol(smcircle_hollow) mcolor(blue)), ///

title("Placebo Test") ///

xlabel(-2(1)2) ylabel(,angle(0)) ///

xline(0.434, lwidth(vthin) lp(shortdash)) xtitle("Coefficients") ///

yline(0.1,lwidth(vthin) lp(dash)) ytitle(p value) ///

legend(label(1 "kdensity of estimates") label( 2 "p value")) ///

plotregion(style(none)) ///无边框

graphregion(color(white)) //白底

*基层医疗机构人均诊疗人次

clear

use "C:\S4 File. Data sample.dta"

set seed 0001

gen tmp = runiform() //生成随机数

sort tmp

psmatch2 ifygt agingdegree1 sexrate urpopustr covid_19 lngdp tc0015100,logit ate neighbor(1) common caliper(.05) ties

pstest agingdegree1 sexrate urpopustr covid_19 lngdp tc0015100,both graph

drop if _weight ==.

reghdfe tf0045103 ygtdid agingdegree1 urpopustr lngdp, absorb(id year) vce(r) //基层人均诊疗人次 显著正向

duplicates report id year //检查个体-年份是否能唯一识别

duplicates example id year //举例子看哪里数据重复

duplicates drop id year,force //强制删除重复数据

tsset id year //再次设置面板数据

xtdes //查看数据

**生成储存每次循环估计系数、标准误和p值的矩阵

set matsize 999

mat b =J(500,1,0) //系数矩阵

mat se = J(500,1,0) //标准误矩阵

mat p = J(500,1,0) //p值矩阵

**循环500次抽取随机样本并回归

forvalues i = 1/500{

use C:\S4 File. Data sample.dta, clear

**设置面板数据

***由于该数据集中有重复数据，先删去重复数据

duplicates report id year //检查个体-年份是否能唯一识别

duplicates example id year //举例子看哪里数据重复

duplicates drop id year,force //强制删除重复数据

xtset id year //设置面板数据

keep if year ==2015 //保留一期数据

sample 37, count //随机抽取326家快餐店（处理组样本容量为326，可从平衡性分析结果看出）

keep id //得到抽取样本id编号

save match_id.dta, replace //另存id数据

merge 1:m id using "C:\S4 File. Data sample.dta" //与原数据匹配

drop ifygt ygtdid //删掉原本的处理组虚拟变量

gen ifygt = (_merge == 3) //将所抽取样本赋值为1，其余为0，得到伪处理虚拟变量

gen ygtdid =ifygt*ygtt

quiet reghdfe tf0045103 ygtdid agingdegree1 urpopustr lngdp, absorb(id year) vce(r)

* 将回归结果赋值到对应矩阵的对应位置

mat b[`i',1] = _b[ygtdid]

mat se[`i',1] = _se[ygtdid]

* 计算P值并赋值于矩阵

mat p[`i',1] = 2*ttail(e(df_r), abs(_b[ygtdid]/_se[ygtdid]))

}

* 矩阵转化为向量

svmat b, names(coef)

svmat se, names(se)

svmat p, names(pvalue)

* 删除空值并添加标签

drop if pvalue1 == .

label var pvalue1 p值

label var coef1 估计系数

keep coef1 se1 pvalue1

save placebo.dta,replace

*绘图

use placebo.dta,clear

twoway (kdensity coef1) (scatter pvalue1 coef1, msymbol(smcircle_hollow) mcolor(blue)), ///

title("Placebo Test") ///

xlabel(-2(1)2) ylabel(,angle(0)) ///

xline(0.340, lwidth(vthin) lp(shortdash)) xtitle("Coefficients") ///

yline(0.1,lwidth(vthin) lp(dash)) ytitle(p value) ///

legend(label(1 "kdensity of estimates") label( 2 "p value")) ///

plotregion(style(none)) ///无边框

graphregion(color(white)) //白底

*Number of hospitalizations per capita

clear

use "C:\S4 File. Data sample.dta"

set seed 0001

gen tmp = runiform() //生成随机数

sort tmp

psmatch2 ifygt agingdegree1 sexrate urpopustr covid_19 lngdp tc0015100,logit ate neighbor(1) common caliper(.05) ties

pstest agingdegree1 sexrate urpopustr covid_19 lngdp tc0015100,both graph

drop if _weight ==.

reghdfe h0 ygtdid agingdegree1 urpopustr lngdp, absorb(id year) vce(r) //人均入院人次 显著正向

duplicates report id year //检查个体-年份是否能唯一识别

duplicates example id year //举例子看哪里数据重复

duplicates drop id year,force //强制删除重复数据

tsset id year //再次设置面板数据

xtdes //查看数据

**生成储存每次循环估计系数、标准误和p值的矩阵

set matsize 999

mat b =J(500,1,0) //系数矩阵

mat se = J(500,1,0) //标准误矩阵

mat p = J(500,1,0) //p值矩阵

**循环500次抽取随机样本并回归

forvalues i = 1/500{

use C:\S4 File. Data sample.dta, clear

**设置面板数据

***由于该数据集中有重复数据，先删去重复数据

duplicates report id year //检查个体-年份是否能唯一识别

duplicates example id year //举例子看哪里数据重复

duplicates drop id year,force //强制删除重复数据

xtset id year //设置面板数据

keep if year ==2015 //保留一期数据

sample 37, count //随机抽取326家快餐店（处理组样本容量为326，可从平衡性分析结果看出）

keep id //得到抽取样本id编号

save match_id.dta, replace //另存id数据

merge 1:m id using "C:\S4 File. Data sample.dta" //与原数据匹配

drop ifygt ygtdid //删掉原本的处理组虚拟变量

gen ifygt = (_merge == 3) //将所抽取样本赋值为1，其余为0，得到伪处理虚拟变量

gen ygtdid =ifygt*ygtt

quiet reghdfe h0 ygtdid agingdegree1 urpopustr lngdp, absorb(id year) vce(r)

* 将回归结果赋值到对应矩阵的对应位置

mat b[`i',1] = _b[ygtdid]

mat se[`i',1] = _se[ygtdid]

* 计算P值并赋值于矩阵

mat p[`i',1] = 2*ttail(e(df_r), abs(_b[ygtdid]/_se[ygtdid]))

}

* 矩阵转化为向量

svmat b, names(coef)

svmat se, names(se)

svmat p, names(pvalue)

* 删除空值并添加标签

drop if pvalue1 == .

label var pvalue1 p值

label var coef1 估计系数

keep coef1 se1 pvalue1

save placebo.dta,replace

*绘图

use placebo.dta,clear

twoway (kdensity coef1) (scatter pvalue1 coef1, msymbol(smcircle_hollow) mcolor(blue)), ///

title("Placebo Test") ///

xlabel(-0.05(0.01)0.05) ylabel(,angle(0)) ///

xline(0.020, lwidth(vthin) lp(shortdash)) xtitle("Coefficients") ///

yline(0.1,lwidth(vthin) lp(dash)) ytitle(p value) ///

legend(label(1 "kdensity of estimates") label( 2 "p value")) ///

plotregion(style(none)) ///无边框

graphregion(color(white)) //白底

*公立医院人均入院人次

clear

use "C:\S4 File. Data sample.dta"

set seed 0001

gen tmp = runiform() //生成随机数

sort tmp

psmatch2 ifygt agingdegree1 sexrate urpopustr covid_19 lngdp tc0015100,logit ate neighbor(1) common caliper(.05) ties

pstest agingdegree1 sexrate urpopustr covid_19 lngdp tc0015100,both graph

drop if _weight ==.

quiet reghdfe tf0071100 ygtdid agingdegree1 urpopustr lngdp, absorb(id year) vce(r) //公立医院人均入院人次 显著正向

duplicates report id year //检查个体-年份是否能唯一识别

duplicates example id year //举例子看哪里数据重复

duplicates drop id year,force //强制删除重复数据

tsset id year //再次设置面板数据

xtdes //查看数据

**生成储存每次循环估计系数、标准误和p值的矩阵

set matsize 999

mat b =J(500,1,0) //系数矩阵

mat se = J(500,1,0) //标准误矩阵

mat p = J(500,1,0) //p值矩阵

**循环500次抽取随机样本并回归

forvalues i = 1/500{

use C:\S4 File. Data sample.dta, clear

**设置面板数据

***由于该数据集中有重复数据，先删去重复数据

duplicates report id year //检查个体-年份是否能唯一识别

duplicates example id year //举例子看哪里数据重复

duplicates drop id year,force //强制删除重复数据

xtset id year //设置面板数据

keep if year ==2015 //保留一期数据

sample 37, count //随机抽取326家快餐店（处理组样本容量为326，可从平衡性分析结果看出）

keep id //得到抽取样本id编号

save match_id.dta, replace //另存id数据

merge 1:m id using "C:\S4 File. Data sample.dta" //与原数据匹配

drop ifygt ygtdid //删掉原本的处理组虚拟变量

gen ifygt = (_merge == 3) //将所抽取样本赋值为1，其余为0，得到伪处理虚拟变量

gen ygtdid =ifygt*ygtt

quiet reghdfe tf0071100 ygtdid agingdegree1 urpopustr lngdp, absorb(id year) vce(r)

* 将回归结果赋值到对应矩阵的对应位置

mat b[`i',1] = _b[ygtdid]

mat se[`i',1] = _se[ygtdid]

* 计算P值并赋值于矩阵

mat p[`i',1] = 2*ttail(e(df_r), abs(_b[ygtdid]/_se[ygtdid]))

}

* 矩阵转化为向量

svmat b, names(coef)

svmat se, names(se)

svmat p, names(pvalue)

* 删除空值并添加标签

drop if pvalue1 == .

label var pvalue1 p值

label var coef1 估计系数

keep coef1 se1 pvalue1

save placebo.dta,replace

*绘图

use placebo.dta,clear

twoway (kdensity coef1) (scatter pvalue1 coef1, msymbol(smcircle_hollow) mcolor(blue)), ///

title("Placebo Test") ///

xlabel(-0.02(0.01)0.02) ylabel(,angle(0)) ///

xline(0.011, lwidth(vthin) lp(shortdash)) xtitle("Coefficients") ///

yline(0.1,lwidth(vthin) lp(dash)) ytitle(p value) ///

legend(label(1 "kdensity of estimates") label( 2 "p value")) ///

plotregion(style(none)) ///无边框

graphregion(color(white)) //白底

*基层医疗机构人均入院人次

clear

use "C:\S4 File. Data sample.dta"

set seed 0001

gen tmp = runiform() //生成随机数

sort tmp

psmatch2 ifygt agingdegree1 sexrate urpopustr covid_19 lngdp tc0015100,logit ate neighbor(1) common caliper(.05) ties

pstest agingdegree1 sexrate urpopustr covid_19 lngdp tc0015100,both graph

drop if _weight ==.

reghdfe tf0045104 ygtdid agingdegree1 urpopustr lngdp, absorb(id year) vce(r) //基层医疗机构人均入院人次 显著正向

duplicates report id year //检查个体-年份是否能唯一识别

duplicates example id year //举例子看哪里数据重复

duplicates drop id year,force //强制删除重复数据

tsset id year //再次设置面板数据

xtdes //查看数据

**生成储存每次循环估计系数、标准误和p值的矩阵

set matsize 999

mat b =J(500,1,0) //系数矩阵

mat se = J(500,1,0) //标准误矩阵

mat p = J(500,1,0) //p值矩阵

**循环500次抽取随机样本并回归

forvalues i = 1/500{

use C:\S4 File. Data sample.dta, clear

**设置面板数据

***由于该数据集中有重复数据，先删去重复数据

duplicates report id year //检查个体-年份是否能唯一识别

duplicates example id year //举例子看哪里数据重复

duplicates drop id year,force //强制删除重复数据

xtset id year //设置面板数据

keep if year ==2015 //保留一期数据

sample 37, count //随机抽取326家快餐店（处理组样本容量为326，可从平衡性分析结果看出）

keep id //得到抽取样本id编号

save match_id.dta, replace //另存id数据

merge 1:m id using "C:\S4 File. Data sample.dta" //与原数据匹配

drop ifygt ygtdid //删掉原本的处理组虚拟变量

gen ifygt = (_merge == 3) //将所抽取样本赋值为1，其余为0，得到伪处理虚拟变量

gen ygtdid =ifygt*ygtt

quiet reghdfe tf0045104 ygtdid agingdegree1 urpopustr lngdp, absorb(id year) vce(r)

* 将回归结果赋值到对应矩阵的对应位置

mat b[`i',1] = _b[ygtdid]

mat se[`i',1] = _se[ygtdid]

* 计算P值并赋值于矩阵

mat p[`i',1] = 2*ttail(e(df_r), abs(_b[ygtdid]/_se[ygtdid]))

}

* 矩阵转化为向量

svmat b, names(coef)

svmat se, names(se)

svmat p, names(pvalue)

* 删除空值并添加标签

drop if pvalue1 == .

label var pvalue1 p值

label var coef1 估计系数

keep coef1 se1 pvalue1

save placebo.dta,replace

*绘图

use placebo.dta,clear

twoway (kdensity coef1) (scatter pvalue1 coef1, msymbol(smcircle_hollow) mcolor(blue)), ///

title("Placebo Test") ///

xlabel(-0.02(0.01)0.02) ylabel(,angle(0)) ///

xline(0.009, lwidth(vthin) lp(shortdash)) xtitle("Coefficients") ///

yline(0.1,lwidth(vthin) lp(dash)) ytitle(p value) ///

legend(label(1 "kdensity of estimates") label( 2 "p value")) ///

plotregion(style(none)) ///无边框

graphregion(color(white)) //白底

*Surgery rate in hospitalized patients

clear

use "C:\S4 File. Data sample.dta"

set seed 0001

gen tmp = runiform() //生成随机数

sort tmp

psmatch2 ifygt agingdegree1 sexrate urpopustr covid_19 lngdp tc0015100,logit ate neighbor(1) common caliper(.05) ties

pstest agingdegree1 sexrate urpopustr covid_19 lngdp tc0015100,both graph

drop if _weight ==.

reghdfe tf0070102 ygtdid agingdegree1 urpopustr lngdp, absorb(id year) vce(r) //医院住院病人手术率 显著正向

duplicates report id year //检查个体-年份是否能唯一识别

duplicates example id year //举例子看哪里数据重复

duplicates drop id year,force //强制删除重复数据

tsset id year //再次设置面板数据

xtdes //查看数据

**生成储存每次循环估计系数、标准误和p值的矩阵

set matsize 999

mat b =J(500,1,0) //系数矩阵

mat se = J(500,1,0) //标准误矩阵

mat p = J(500,1,0) //p值矩阵

**循环500次抽取随机样本并回归

forvalues i = 1/500{

use C:\S4 File. Data sample.dta, clear

**设置面板数据

***由于该数据集中有重复数据，先删去重复数据

duplicates report id year //检查个体-年份是否能唯一识别

duplicates example id year //举例子看哪里数据重复

duplicates drop id year,force //强制删除重复数据

xtset id year //设置面板数据

keep if year ==2015 //保留一期数据

sample 37, count //随机抽取326家快餐店（处理组样本容量为326，可从平衡性分析结果看出）

keep id //得到抽取样本id编号

save match_id.dta, replace //另存id数据

merge 1:m id using "C:\S4 File. Data sample.dta" //与原数据匹配

drop ifygt ygtdid //删掉原本的处理组虚拟变量

gen ifygt = (_merge == 3) //将所抽取样本赋值为1，其余为0，得到伪处理虚拟变量

gen ygtdid =ifygt*ygtt

quiet reghdfe tf0070102 ygtdid agingdegree1 urpopustr lngdp, absorb(id year) vce(r)

* 将回归结果赋值到对应矩阵的对应位置

mat b[`i',1] = _b[ygtdid]

mat se[`i',1] = _se[ygtdid]

* 计算P值并赋值于矩阵

mat p[`i',1] = 2*ttail(e(df_r), abs(_b[ygtdid]/_se[ygtdid]))

}

* 矩阵转化为向量

svmat b, names(coef)

svmat se, names(se)

svmat p, names(pvalue)

* 删除空值并添加标签

drop if pvalue1 == .

label var pvalue1 p值

label var coef1 估计系数

keep coef1 se1 pvalue1

save placebo.dta,replace

*绘图

use placebo.dta,clear

twoway (kdensity coef1) (scatter pvalue1 coef1, msymbol(smcircle_hollow) mcolor(blue)), ///

title("Placebo Test") ///

xlabel(-0.1(0.05)0.1) ylabel(,angle(0)) ///

xline(0.044, lwidth(vthin) lp(shortdash)) xtitle("Coefficients") ///

yline(0.1,lwidth(vthin) lp(dash)) ytitle(p value) ///

legend(label(1 "kdensity of estimates") label( 2 "p value")) ///

plotregion(style(none)) ///无边框

graphregion(color(white)) //白底

*公立医院住院病人手术率

clear

use "C:\S4 File. Data sample.dta"

set seed 0001

gen tmp = runiform() //生成随机数

sort tmp

psmatch2 ifygt agingdegree1 sexrate urpopustr covid_19 lngdp tc0015100,logit ate neighbor(1) common caliper(.05) ties

pstest agingdegree1 sexrate urpopustr covid_19 lngdp tc0015100,both graph

drop if _weight ==.

quiet reghdfe tf0071102 ygtdid agingdegree1 urpopustr lngdp, absorb(id year) vce(r) //公立医院住院病人手术率 显著正向

duplicates report id year //检查个体-年份是否能唯一识别

duplicates example id year //举例子看哪里数据重复

duplicates drop id year,force //强制删除重复数据

tsset id year //再次设置面板数据

xtdes //查看数据

**生成储存每次循环估计系数、标准误和p值的矩阵

set matsize 999

mat b =J(500,1,0) //系数矩阵

mat se = J(500,1,0) //标准误矩阵

mat p = J(500,1,0) //p值矩阵

**循环500次抽取随机样本并回归

forvalues i = 1/500{

use C:\S4 File. Data sample.dta, clear

**设置面板数据

***由于该数据集中有重复数据，先删去重复数据

duplicates report id year //检查个体-年份是否能唯一识别

duplicates example id year //举例子看哪里数据重复

duplicates drop id year,force //强制删除重复数据

xtset id year //设置面板数据

keep if year ==2015 //保留一期数据

sample 37, count //随机抽取326家快餐店（处理组样本容量为326，可从平衡性分析结果看出）

keep id //得到抽取样本id编号

save match_id.dta, replace //另存id数据

merge 1:m id using "C:\S4 File. Data sample.dta" //与原数据匹配

drop ifygt ygtdid //删掉原本的处理组虚拟变量

gen ifygt = (_merge == 3) //将所抽取样本赋值为1，其余为0，得到伪处理虚拟变量

gen ygtdid =ifygt*ygtt

quiet reghdfe tf0071102 ygtdid agingdegree1 urpopustr lngdp, absorb(id year) vce(r)

* 将回归结果赋值到对应矩阵的对应位置

mat b[`i',1] = _b[ygtdid]

mat se[`i',1] = _se[ygtdid]

* 计算P值并赋值于矩阵

mat p[`i',1] = 2*ttail(e(df_r), abs(_b[ygtdid]/_se[ygtdid]))

}

* 矩阵转化为向量

svmat b, names(coef)

svmat se, names(se)

svmat p, names(pvalue)

* 删除空值并添加标签

drop if pvalue1 == .

label var pvalue1 p值

label var coef1 估计系数

keep coef1 se1 pvalue1

save placebo.dta,replace

*绘图

use placebo.dta,clear

twoway (kdensity coef1) (scatter pvalue1 coef1, msymbol(smcircle_hollow) mcolor(blue)), ///

title("Placebo Test") ///

xlabel(-0.1(0.05)0.1) ylabel(,angle(0)) ///

xline(0.050, lwidth(vthin) lp(shortdash)) xtitle("Coefficients") ///

yline(0.1,lwidth(vthin) lp(dash)) ytitle(p value) ///

legend(label(1 "kdensity of estimates") label( 2 "p value")) ///

plotregion(style(none)) ///无边框

graphregion(color(white)) //白底

*Number of beds per thousand people

*社区卫生服务站床位数

clear

use "C:\S4 File. Data sample.dta"

set seed 0001

gen tmp = runiform() //生成随机数

sort tmp

psmatch2 ifygt agingdegree1 sexrate urpopustr covid_19 lngdp tc0015100,logit ate neighbor(1) common caliper(.05) ties

pstest agingdegree1 sexrate urpopustr covid_19 lngdp tc0015100,both graph

drop if _weight ==.

reghdfe td0022109 ygtdid agingdegree1 urpopustr lngdp, absorb(id year) vce(r) //社区卫生服务站床位数 显著正向

duplicates report id year //检查个体-年份是否能唯一识别

duplicates example id year //举例子看哪里数据重复

duplicates drop id year,force //强制删除重复数据

tsset id year //再次设置面板数据

xtdes //查看数据

**生成储存每次循环估计系数、标准误和p值的矩阵

set matsize 999

mat b =J(500,1,0) //系数矩阵

mat se = J(500,1,0) //标准误矩阵

mat p = J(500,1,0) //p值矩阵

**循环500次抽取随机样本并回归

forvalues i = 1/500{

use clear

use C:\S4 File. Data sample.dta, clear

**设置面板数据

***由于该数据集中有重复数据，先删去重复数据

duplicates report id year //检查个体-年份是否能唯一识别

duplicates example id year //举例子看哪里数据重复

duplicates drop id year,force //强制删除重复数据

xtset id year //设置面板数据

keep if year ==2015 //保留一期数据

sample 37, count //随机抽取326家快餐店（处理组样本容量为326，可从平衡性分析结果看出）

keep id //得到抽取样本id编号

save match_id.dta, replace //另存id数据

merge 1:m id using clear

use "C:\S4 File. Data sample.dta"//与原数据匹配

drop ifygt ygtdid //删掉原本的处理组虚拟变量

gen ifygt = (_merge == 3) //将所抽取样本赋值为1，其余为0，得到伪处理虚拟变量

gen ygtdid =ifygt*ygtt

quiet reghdfe td0022109 ygtdid agingdegree1 urpopustr lngdp, absorb(id year) vce(r)

* 将回归结果赋值到对应矩阵的对应位置

mat b[`i',1] = _b[ygtdid]

mat se[`i',1] = _se[ygtdid]

* 计算P值并赋值于矩阵

mat p[`i',1] = 2*ttail(e(df_r), abs(_b[ygtdid]/_se[ygtdid]))

}

* 矩阵转化为向量

svmat b, names(coef)

svmat se, names(se)

svmat p, names(pvalue)

* 删除空值并添加标签

drop if pvalue1 == .

label var pvalue1 p值

label var coef1 估计系数

keep coef1 se1 pvalue1

save placebo.dta,replace

*绘图

use placebo.dta,clear

twoway (kdensity coef1) (scatter pvalue1 coef1, msymbol(smcircle_hollow) mcolor(blue)), ///

title("Placebo Test") ///

xlabel(-3(1)3) ylabel(,angle(0)) ///

xline(1.678, lwidth(vthin) lp(shortdash)) xtitle("Coefficients") ///

yline(0.1,lwidth(vthin) lp(dash)) ytitle(p value) ///

legend(label(1 "kdensity of estimates") label( 2 "p value")) ///

plotregion(style(none)) ///无边框

graphregion(color(white)) //白底

clear

use "C:\S4 File. Data sample.dta"

gen ygtt=0

replace ygtt=1 if year>=2019

g ygtdid=ygtt*ifygt

set seed 0001

gen tmp = runiform() //生成随机数

sort tmp

psmatch2 ifygt agingdegree1 sexrate urpopustr covid_19 lngdp , logit ate neighbor(1) common caliper(.05) ties

pstest agingdegree1 sexrate urpopustr covid_19 lngdp ,both graph

drop if _weight ==.

*Robustness test

*Diversion Effect

*PSM

use "C:\S4 File. Data sample.dta"

drop if year<2016

gen ygtt=0

replace ygtt=1 if year>=2019

g ygtdid=ygtt*ifygt

set seed 0001

gen tmp = runiform() //生成随机数

sort tmp

psmatch2 ifygt agingdegree1 sexrate urpopustr covid_19 lngdp tc0015100,logit ate neighbor(1) common caliper(.05) ties

pstest agingdegree1 sexrate urpopustr covid_19 lngdp tc0015100,both graph

drop if _weight ==.

*DID-Number of consultations per capita(dtp)

reghdfe y0 ygtdid agingdegree1 urpopustr lngdp, absorb(id year) vce(r) //人均诊疗人次 显著正向

est sto s1

reghdfe tf0066100 ygtdid agingdegree1 urpopustr lngdp, absorb(id year) vce(r) //公立医院诊疗人次

est sto s2

reghdfe tf0045103 ygtdid agingdegree1 urpopustr lngdp, absorb(id year) vce(r) //基层医疗机构诊疗人次 显著正向

est sto s3

esttab s1 s2 s3 ,ar2(%8.4f)se(%8.4f) star(* 0.1 ** 0.05 *** 0.01) aic bic mtitles

outreg2[s1 s2 s3] using Robustness test Diversion Effect(dtp).doc, bdec(3) sdec(3) addtext(year FE,yes,city FE,yes)

*DID-The proportion of consultations in different levels of medical institutions(dtr)

reghdfe y3 ygtdid agingdegree1 urpopustr lngdp, absorb(id year) vce(r) //公立医院诊疗人次占比

est sto s1

reghdfe y1 ygtdid agingdegree1 urpopustr lngdp, absorb(id year) vce(r) //基层医疗机构诊疗人次占比

est sto s2

esttab s1 s2,ar2(%8.4f)se(%8.4f) star(* 0.1 ** 0.05 *** 0.01) aic bic mtitles

outreg2[s1 s2 ] using Robustness test Diversion Effect(dtr).doc, bdec(3) sdec(3) addtext(year FE,yes,city FE,yes)

*Diagnosis and treatment effect

*PSM

clear

use "C:\S4 File. Data sample.dta"

drop if year<2016

gen ygtt=0

replace ygtt=1 if year>=2019

g ygtdid=ygtt*ifygt

set seed 0001

gen tmp = runiform() //生成随机数

sort tmp

psmatch2 ifygt agingdegree1 sexrate urpopustr covid_19 lngdp tc0015100,logit ate neighbor(1) common caliper(.05) ties

pstest agingdegree1 sexrate urpopustr covid_19 lngdp tc0015100,both graph

drop if _weight ==.

*Diagnosis and treatment effect-Number of hospitalizations per capita (htp)

reghdfe h0 ygtdid agingdegree1 urpopustr lngdp, absorb(id year) vce(r) //人均入院人次 显著正向

est sto s1

reghdfe tf0071100 ygtdid agingdegree1 urpopustr lngdp, absorb(id year) vce(r) //公立入院人次 显著正向

est sto s2

reghdfe tf0045104 ygtdid agingdegree1 urpopustr lngdp, absorb(id year) vce(r) //基层医疗机构入院人次 did显著正向

est sto s3

esttab s1 s2 s3 ,ar2(%8.4f)se(%8.4f) star(* 0.1 ** 0.05 *** 0.01) aic bic mtitles

outreg2[s1 s2 s3 ] using Robustness test (htp).doc, bdec(3) sdec(3) addtext(year FE,yes,city FE,yes)

*Diagnosis and treatment effect-The proportion of inpatients in different levels of medical institutions(htr)

reghdfe h3 ygtdid agingdegree1 urpopustr lngdp, absorb(id year) vce(r) //公立入院人次占比

est sto s1

reghdfe h1 ygtdid agingdegree1 urpopustr lngdp, absorb(id year) vce(r) //基层医疗机构入院人次占比 did显著正向

est sto s2

esttab s1 s2 ,ar2(%8.4f)se(%8.4f) star(* 0.1 ** 0.05 *** 0.01) aic bic mtitles

outreg2[s1 s2 ] using Robustness test(htr).doc, bdec(3) sdec(3) addtext(year FE,yes,city FE,yes)

*Diagnosis and treatment effect-Surgery rate in hospitalized patients(htsr)

reghdfe tf0070102 ygtdid agingdegree1 urpopustr lngdp, absorb(id year) vce(r) //医院住院病人手术率 显著正向

est sto s1

reghdfe tf0071102 ygtdid agingdegree1 urpopustr lngdp, absorb(id year) vce(r) //公立住院病人手术率 显著正向

est sto s2

esttab s1 s2 ,ar2(%8.4f)se(%8.4f) star(* 0.1 ** 0.05 *** 0.01) aic bic mtitles

outreg2[s1 s2 ] using Robustness test(htr).doc, bdec(3) sdec(3) addtext(year FE,yes,city FE,yes)

*Health workers per thousand people(pher)

clear

use "C:\S4 File. Data sample.dta"

drop if year<2016

set seed 0001

gen tmp = runiform() //生成随机数

sort tmp

psmatch2 ifygt agingdegree1 sexrate urpopustr covid_19 lngdp ,outcome(tc0015100) logit ate neighbor(1) common caliper(.05) ties

pstest agingdegree1 sexrate urpopustr covid_19 lngdp ,both graph

drop if _weight ==.

gen ygtt=0

replace ygtt=1 if year>=2019

g ygtdid=ygtt*ifygt

reghdfe tc0015100 ygtdid agingdegree1 lngdp, absorb(id year) vce(r) //医院卫生技术人员

est sto s1

esttab s1 ,ar2(%8.4f)se(%8.4f) star(* 0.1 ** 0.05 *** 0.01) aic bic mtitles

outreg2[s1 ] using Robustness test(pher)-1.doc, bdec(3) sdec(3) addtext(year FE,yes,city FE,yes)

clear

use "C:\S4 File. Data sample.dta"

drop if year<2016

set seed 0001

gen tmp = runiform() //生成随机数

sort tmp

psmatch2 ifygt agingdegree1 sexrate urpopustr covid_19 lngdp ,outcome(tc0016101) logit ate neighbor(1) common caliper(.05) ties

pstest agingdegree1 sexrate urpopustr covid_19 lngdp ,both graph

drop if _weight ==.

gen ygtt=0

replace ygtt=1 if year>=2019

g ygtdid=ygtt*ifygt

reghdfe tc0016101 ygtdid agingdegree1 sexrate urpopustr lngdp, absorb(id year) vce(r) //乡镇卫生院卫生技术人员

est sto s1

esttab s1 ,ar2(%8.4f)se(%8.4f) star(* 0.1 ** 0.05 *** 0.01) aic bic mtitles

outreg2[s1] using Robustness test(pher)-2.doc, bdec(3) sdec(3) addtext(year FE,yes,city FE,yes)

clear

use "C:\S4 File. Data sample.dta"

drop if year<2016

set seed 0001

gen tmp = runiform() //生成随机数

sort tmp

psmatch2 ifygt agingdegree1 sexrate urpopustr covid_19 lngdp ,outcome(tc0017101) logit ate neighbor(1) common caliper(.05) ties

pstest agingdegree1 sexrate urpopustr covid_19 lngdp ,both graph

drop if _weight ==.

gen ygtt=0

replace ygtt=1 if year>=2019

g ygtdid=ygtt*ifygt

reghdfe tc0017100 ygtdid sexrate lngdp, absorb(id year) vce(r) //社区卫生服务中心卫生技术人员

est sto s1

esttab s1 ,ar2(%8.4f)se(%8.4f) star(* 0.1 ** 0.05 *** 0.01) aic bic mtitles

outreg2[s1] using Robustness test(pher)-3.doc, bdec(3) sdec(3) addtext(year FE,yes,city FE,yes)

*Resource allocation effect

*Number of beds per thousand people(phbed)

reghdfe td0022101 ygtdid agingdegree1 lngdp , absorb(id year) vce(r) //公立医院小计

est sto s0

reghdfe td0022108 ygtdid agingdegree1 lngdp , absorb(id year) vce(r) //社区卫生服务中心

est sto s1

reghdfe td0022109 ygtdid agingdegree1 lngdp , absorb(id year) vce(r) //社区卫生服务站 显著正向

est sto s2

reghdfe td0022111 ygtdid agingdegree1 lngdp , absorb(id year) vce(r) //乡镇卫生院

est sto s3

reghdfe td0022112 ygtdid agingdegree1 lngdp , absorb(id year) vce(r) //门诊部 显著负向

est sto s4

esttab s0 s1 s2 s3 s4 ,ar2(%8.4f)se(%8.4f) star(* 0.1 ** 0.05 *** 0.01) aic bic mtitles

outreg2[s0 s1 s2 s3 s4] using Robustness test(phbed).doc, bdec(3) sdec(3) addtext(year FE,yes,city FE,yes)
