# Supplementary material for: County medical community, medical insurance package payment, and hierarchical diagnosis and treatment—Empirical analysis of the impact of the pilot project of compact county medical communities in Sichuan Province
Source: PLoS One. 2024 Apr 5;19(4):e0297340. doi: 10.1371/journal.pone.0297340 (PMC10997099; doi:10.1371/journal.pone.0297340)
Supplement: Attachment — Submitted filename: PONE-D-23-37259-AF.pdf [file pone.0297340.s005.pdf]

PLOS ONE

County Medical Community, Medical Insurance Package Payment, and Hierarchical Diagnosis and Treatment

--Manuscript Draft--

**Manuscript Number:**	PONE-D-23-37259
**Article Type:**	Research Article
**Full Title:**	County Medical Community, Medical Insurance Package Payment, and Hierarchical Diagnosis and Treatment
**Short Title:**	Empirical Analysis of the Pilot Impact of Compact County Medical Communities in Sichuan Province
**Corresponding Author:**	Yuxuan ZHOU, ph.D<br>Southwestern University of Finance and Economics<br>Chengdu, CHINA
**Keywords:**	Comprehensive Medical Insurance Prepayment; Medical Community; Hierarchical diagnosis and treatment
**Abstract:**	Hierarchical diagnosis and treatment is an important exploration direction to alleviate the rising pressure of health expenses and medical insurance fund expenditure in China, and to maintain and protect the health of the broad masses of the people. In recent years, the construction of compact county medical community has become the main starting point of hierarchical diagnosis and treatment reform. This paper uses the quasi-natural experiment of the pilot of the compact county medical community in Sichuan Province in 2019 and the county-level data of the ' Sichuan Provincial Health Statistics Yearbook ' from 2008 to 2021 to evaluate the effect of the pilot of the medical community on promoting hierarchical diagnosis and treatment under the medical insurance package payment model. The results show that the pilot of county medical community has significantly increased the number of per capita diagnosis and treatment of medical and health institutions in pilot districts and counties by 0.434 times, of which the number of per capita diagnosis and treatment of primary medical institutions has increased by 0.340 times ; the number of hospitalizations per capita in public hospitals and primary medical institutions in pilot districts and counties increased significantly, and the operation rate of inpatients in public hospitals increased by 5 %. There was no significant impact on the allocation of medical facilities and human resources in the pilot counties. Therefore, the construction of county medical community under the medical insurance package payment mode has promoted the realization of county hierarchical diagnosis and treatment pattern.
**Order of Authors:**	Shaoqun DING, ph.D
	Yuxuan ZHOU, ph.D
**Opposed Reviewers:**	
**Additional Information:**	
**Question**	**Response**
**Financial Disclosure**<br><br>Enter a financial disclosure statement that describes the sources of funding for the work included in this submission. Review the submission guidelines for detailed requirements. View published research articles from *PLOS ONE* for specific examples.	Yes

Powered by Editorial Manager® and ProduXion Manager® from Aries Systems Corporation

This statement is required for submission and **will appear in the published article** if the submission is accepted. Please make sure it is accurate.<br><br>**Funded studies**<br>Enter a statement with the following details:<br>• Initials of the authors who received each award<br>• Grant numbers awarded to each author<br>• The full name of each funder<br>• URL of each funder website<br>• Did the sponsors or funders play any role in the study design, data collection and analysis, decision to publish, or preparation of the manuscript?<br><br>Did you receive funding for this work?	
Please add funding details.<br>as follow-up to "**Financial Disclosure**<br><br>Enter a financial disclosure statement that describes the sources of funding for the work included in this submission. Review the submission guidelines for detailed requirements. View published research articles from *PLOS ONE* for specific examples.<br><br>This statement is required for submission and **will appear in the published article** if the submission is accepted. Please make sure it is accurate.<br><br>**Funded studies**<br>Enter a statement with the following details:<br>• Initials of the authors who received each award<br>• Grant numbers awarded to each author<br>• The full name of each funder<br>• URL of each funder website<br>• Did the sponsors or funders play any role in the study design, data collection and analysis, decision to publish, or preparation of the manuscript?<br><br>Did you receive funding for this work?"	National Social Science Fund Major Project ' Research on Multi-level Social Security System Innovation and Policy Synergy Based on System Concept ' (23ZDA099) ; the basic scientific research business fee of the central university ' Research on the effect of urban and rural residents ' medical insurance outpatient co-ordination to promote hierarchical diagnosis and treatment ' graduate research project (JBK2307025).

Powered by Editorial Manager® and ProduXion Manager® from Aries Systems Corporation

Please select the country of your main research funder (please select carefully as in some cases this is used in fee calculation).<br>as follow-up to "**Financial Disclosure**<br><br>Enter a financial disclosure statement that describes the sources of funding for the work included in this submission. Review the submission guidelines for detailed requirements. View published research articles from *PLOS ONE* for specific examples.<br><br>This statement is required for submission and **will appear in the published article** if the submission is accepted. Please make sure it is accurate.<br><br>**Funded studies**<br>Enter a statement with the following details:<br>• Initials of the authors who received each award<br>• Grant numbers awarded to each author<br>• The full name of each funder<br>• URL of each funder website<br>• Did the sponsors or funders play any role in the study design, data collection and analysis, decision to publish, or preparation of the manuscript?<br><br>Did you receive funding for this work?"	CHINA - CN
**Competing Interests**<br><br>Use the instructions below to enter a competing interest statement for this submission. On behalf of all authors, disclose any competing interests that could be perceived to bias this work—acknowledging all financial support and any other relevant financial or non-financial competing interests.<br><br>This statement is required for submission and **will appear in the published article** if the submission is accepted. Please make sure it is accurate and that any funding	The authors have declared that no competing interests exist.

sources listed in your Funding Information later in the submission form are also declared in your Financial Disclosure statement.<br><br>View published research articles from *PLOS ONE* for specific examples.<br><br>**NO authors have competing interests**<br><br>Enter: *The authors have declared that no competing interests exist.*<br><br>**Authors with competing interests**<br><br>Enter competing interest details beginning with this statement:<br><br>*I have read the journal's policy and the authors of this manuscript have the following competing interests: [insert competing interests here]*<br><br>* typeset	
**Ethics Statement**<br><br>Enter an ethics statement for this submission. This statement is required if the study involved:<br><br>• Human participants<br>• Human specimens or tissue<br>• Vertebrate animals or cephalopods<br>• Vertebrate embryos or tissues<br>• Field research<br><br>Write "N/A" if the submission does not require an ethics statement.<br><br>General guidance is provided below. Consult the submission guidelines for detailed instructions. **Make sure that all information entered here is included in the Methods section of the manuscript.**	N/A

**Format for specific study types**<br><br>**Human Subject Research (involving human participants and/or tissue)**<br>• Give the name of the institutional review board or ethics committee that approved the study<br>• Include the approval number and/or a statement indicating approval of this research<br>• Indicate the form of consent obtained (written/oral) or the reason that consent was not obtained (e.g. the data were analyzed anonymously)<br><br>**Animal Research (involving vertebrate animals, embryos or tissues)**<br>• Provide the name of the Institutional Animal Care and Use Committee (IACUC) or other relevant ethics board that reviewed the study protocol, and indicate whether they approved this research or granted a formal waiver of ethical approval<br>• Include an approval number if one was obtained<br>• If the study involved *non-human primates*, add *additional details* about animal welfare and steps taken to ameliorate suffering<br>• If anesthesia, euthanasia, or any kind of animal sacrifice is part of the study, include briefly which substances and/or methods were applied<br><br>**Field Research**<br><br>Include the following details if this study involves the collection of plant, animal, or other materials from a natural setting:<br>• Field permit number<br>• Name of the institution or relevant body that granted permission	
**Data Availability**<br><br>Authors are required to make all data underlying the findings described fully available, without restriction, and from the time of publication. PLOS allows rare exceptions to address legal and ethical concerns. See the PLOS Data Policy and FAQ for detailed information.	Yes - all data are fully available without restriction

Powered by Editorial Manager® and ProduXion Manager® from Aries Systems Corporation

A Data Availability Statement describing where the data can be found is required at submission. Your answers to this question constitute the Data Availability Statement and **will be published in the article**, if accepted.<br><br>**Important:** Stating 'data available on request from the author' is not sufficient. If your data are only available upon request, select 'No' for the first question and explain your exceptional situation in the text box.<br><br>Do the authors confirm that all data underlying the findings described in their manuscript are fully available without restriction?	
**Describe where the data may be found in full sentences. If you are copying our sample text, replace any instances of XXX with the appropriate details.**<br><br>• If the data are **held or will be held in a public repository**, include URLs, accession numbers or DOIs. If this information will only be available after acceptance, indicate this by ticking the box below. For example: *All XXX files are available from the XXX database (accession number(s) XXX, XXX.).*<br>• If the data are all contained **within the manuscript and/or Supporting Information files**, enter the following: *All relevant data are within the manuscript and its Supporting Information files.*<br>• If neither of these applies but you are able to provide **details of access elsewhere**, with or without limitations, please do so. For example:<br><br>*Data cannot be shared publicly because of [XXX]. Data are available from the XXX Institutional Data Access / Ethics Committee (contact via XXX) for researchers who meet the criteria for access to confidential data.*<br><br>*The data underlying the results presented in the study are available from (include the name of the third party*	All relevant data are within the manuscript and its Supporting Information files.

and contact information or URL).<br>• This text is appropriate if the data are owned by a third party and authors do not have permission to share the data.<br><br>* typeset	
Additional data availability information:	

Powered by Editorial Manager® and ProduXion Manager® from Aries Systems Corporation

Dear Editor,

On behalf of all authors, I would like to ask you to consider our manuscript entitled “County Medical Community, Medical Insurance Package Payment, and Hierarchical Diagnosis and Treatment——Empirical Analysis of the Pilot Impact of Compact County Medical Communities in Sichuan Province” for publication in *PLOS ONE* as an original research article.

In recent years, the construction of compact county medical community has become the main starting point of hierarchical diagnosis and treatment reform. This paper evaluate the effect of the pilot of the medical community in Sichuan Province on promoting hierarchical diagnosis and treatment under the medical insurance package payment model.

We find that the construction of county medical community under the medical insurance package payment mode significantly increased the county consultation rate among residents in the pilot areas,especially primary medical care,and enhanced the inpatient operation rate in hospitals at the same time. We believe that findings from this study will be of special interest to the readers of *PLOS ONE*.

Sincerely yours,

Yuxuan ZHOU，Doctoral student

School of Public Administration, Southwestern University of Finance and Economics,Chengdu, Sichuan, China

Current Address: Liulin Campus, Southwestern University of Finance and Economics, 555 Liutai Avenue, Wenjiang District, Chengdu , Sichuan, China

86+15556932867
15556932867@163.com

Manuscript Click here to access/download;Manuscript;Manuscript.docx

County Medical Community, Medical Insurance Package Payment, and Hierarchical Diagnosis and Treatment

——Empirical Analysis of the ~~Pilot Impact~~ of Compact County Medical Communities in Sichuan Province

Shaoqun Ding[1,#a], Yuxuan Zhou[2,#a,*]

[1]Research Center for Aging and Social Security, Southwestern University of Finance and Economics,Chengdu, Sichuan, China

2School of Public Administration,Southwestern University of Finance and Economics,Chengdu, Sichuan, China

#aCurrent Address: Liulin Campus, Southwestern University of Finance and Economics, 555 Liutai Avenue, Wenjiang District, Chengdu , Sichuan, China

* Corresponding author
E-mail:15556932867@163.com(Zhou)

Abstract

Hierarchical diagnosis and treatment is an important exploration direction to alleviate the rising pressure of health expenses and medical insurance fund expenditure in China, and to maintain and protect the health of the broad masses of the people. In recent years, the construction of compact county medical community has become the main starting point of hierarchical diagnosis and treatment reform. This paper uses the quasi-natural experiment of the pilot of the compact county medical community in Sichuan Province in 2019 and the county-level data of the ' Sichuan Provincial Health Statistics Yearbook ' from 2008 to 2021 to evaluate the effect of the pilot of the medical community on promoting hierarchical diagnosis and treatment under the medical insurance package payment model. The results show that the pilot of county medical community has significantly increased the number of per capita diagnosis and treatment of medical and health institutions in pilot districts and counties by 0.434 times, of which the number of per capita diagnosis and treatment of primary medical institutions has increased by 0.340 times ; the number of hospitalizations per capita in public hospitals and primary medical institutions in pilot districts and counties increased significantly, and the operation rate of inpatients in public hospitals increased by 5 %. There was no significant impact on the allocation of medical facilities and human resources in the pilot counties. Therefore, the construction of county medical community under the medical insurance package payment mode has promoted the realization of county hierarchical diagnosis and treatment pattern.

Key words:Comprehensive Medical Insurance Prepayment; Medical Community; Hierarchical diagnosis and treatment

1.Introduction

China's reform of hierarchical diagnosis and treatment primarily relies on the medical consortium model to achieve its goals[1]. The concept of a medical consortium is rooted in the global practice of Integrated Healthcare, which involves the collaboration of multiple medical institutions. By leveraging the complementary strengths and the judicious allocation of resources among these medical service providers, a medical consortium offers patients a seamless continuum of healthcare services, enhancing service quality while reducing costs. This, in turn, seeks to optimize the utilization of medical resources[2].

In 2015, The General Office of the State Council released the "Guiding Opinions on Promoting the Construction of a Hierarchical Diagnosis and Treatment System," which advocated the exploration of medical consortium formation and the gradual development of a regional collaborative service model with clearly defined roles, rights, and benefits. Since the inception of the medical consortium construction in 2015, various pilot areas have ventured into diverse modes of exploration. These include early trusted-type county medical communities, loose medical communities, and loose county medical communities. Notable examples of medical community models include those implemented in Tianchang, Anhui, Funan, Anhui, and Youxi, Fujian.

In April 2017, The General Office of the State Council issued the "Guiding Opinions on Promoting the Construction and Development of Medical Consortia," which introduced the

concept of four distinct medical consortia, namely, city medical groups, county medical consortia, inter-regional specialty alliances, and telemedicine collaboration networks, each with its specific functional roles. The focus was on the exploration of integrated management at the county and township levels, with county-level public hospitals taking the lead, township health centers serving as the central hubs, and village clinics forming the foundational basis. This approach aimed to establish a county-level medical service system characterized by seamless collaboration and coordination across all three levels, from counties down to villages.Consequently, in 2017, pilot regions initiated their efforts to explore the establishment of tightly-knit medical communities.

In June 2017, The General Office of the State Council released the "Guiding Opinions on Further Deepening the Reform of Basic Medical Insurance Payment Methods." This document proposed the exploration of implementing comprehensive medical insurance prepayment for vertically aligned cooperative medical unions, including county medical communities, and other collaborative mechanisms.

In May 2019, the National Health Commission and the State Administration of Traditional Chinese Medicine issued a notice that urged the enhancement of various payment methods, such as comprehensive medical insurance payments. It also encouraged the exploration of total head budget management for medical insurance, along with the establishment of mechanisms to retain surplus funds and share reasonable expenditure.

Subsequently, in August 2020, the National Health Commission and other relevant departments issued a notice introducing the Evaluation Criteria and Monitoring Index System for the Construction of Compact County Medical and Health Communities (Trial). This notice explicitly called for the development of payment policies tailored to the specific characteristics of county medical communities and the exploration of comprehensive payment mechanisms facilitated by the medical insurance fund for these communities.

The reform of the "Medical Insurance Package Payment," a multifaceted and intricate medical insurance payment approach within the overarching comprehensive medical insurance system, plays a pivotal role in providing substantial support for the development of medical communities.

Following the issuance of the Notice on Promoting the Construction of Compact County Medical and Health Communities in 2019, the National Health Commission initiated a nationwide pilot project for the establishment of such communities. It identified 754 counties (cities and districts) in Shanxi and Zhejiang provinces, along with 567 counties (cities and districts) in other provinces as pilot counties. In September 2019, the Sichuan Provincial Health Commission, in collaboration with other relevant departments, released the "Implementation Plan for the Construction of Compact County Medical and Health Communities in Sichuan Province" (referred to as the "Implementation Plan" hereinafter). This plan drew on the earlier nationwide pilot experiences in policy formulation, refining the roles and authority of each system participant.

Differing from the province-wide implementation of the compact county health community pilot in Shanxi and Zhejiang provinces, Sichuan Province, which had not previously conducted such a pilot, selected specific districts and counties to implement the pilot project across the entire

province, providing a valuable experimental group and control group for this study.

As a unified medical organization alliance, the county medical community is anticipated to optimize the integration of medical resources, achieve efficient resource allocation, enhance grassroots service capabilities, and promote the realization of a hierarchical diagnosis and treatment system[3,4].Therefore, the key questions of interest in this paper pertain to the effectiveness of the compact medical community pilot in Sichuan Province in 2019.

Firstly, can the county health community, dedicated to enhancing primary care services, effectively guide patients to seek medical care at the county level?Secondly, the establishment of medical communities has transformed the competitive relationships between medical institutions at all levels into cooperative ones. Will this affect the diagnosis and treatment practices of these institutions at various levels?Finally, can the creation of compact medical communities genuinely improve the allocation of medical resources, including health personnel and medical facilities?

These are crucial questions addressed in this paper. Accordingly, relying on data from the Sichuan Provincial Health Statistical Yearbook spanning from 2008 to 2021, the paper employs the PSM-DID (Propensity Score Matching and Difference-in-Differences) method to assess the effectiveness of the pilot counties in the implementation of compact medical communities in Sichuan Province.

The other sections of this paper are structured as follows:

Background and Literature Review: This section delves into the historical evolution of hierarchical diagnosis and treatment both domestically and internationally. It also provides an overview of the reforms in medical insurance payment methods, the restructuring of medical consortia and medical communities, and the assessments and appraisals by scholars, both national and international, on the establishment of an integrated medical system and the role of medical unions and medical communities.

Theoretical Mechanisms: This segment outlines the optimal decisions made by each participant in the county medical community construction. It explores how these decisions align with the incentive compatibility mechanism of "medical insurance package payment" and the internal reforms of medical communities.

Empirical Analysis: In this part, the paper details the sources of data used, the selection of variables, and the choice of models for analysis. It further presents the empirical findings on various topics of interest within the paper and concludes with policy recommendations and conclusions derived from the empirical results.

The structure of this paper provides a comprehensive examination of the development and implications of hierarchical diagnosis and treatment, with a focus on the implementation of compact medical communities and their effects within the context of evolving medical insurance payment systems.

2.Literature Review

The structure of this paper provides a comprehensive examination of the development and

implications of hierarchical diagnosis and treatment, with a focus on the implementation of compact medical communities and their effects within the context of evolving medical insurance payment systems。

Hierarchical diagnosis and treatment entail ensuring that all patients receive the appropriate care at the right time and in the right healthcare setting. One of its key benefits lies in providing patients with continuous, high-quality care at a relatively low cost[5]. In China, healthcare providers are categorized into two primary tiers: primary medical institutions and hospitals. The former, typically offering general medicine services, primarily handle the diagnosis and treatment of common and frequently encountered health conditions, while the latter, housing specialized departments, focus on diagnosing and treating complex and severe diseases.

From the provider's perspective, the hierarchical diagnosis and treatment system is orchestrated by the health department. This involves the implementation of policies such as enlisting family doctors as gatekeepers, the establishment of medical unions and medical communities, and the reform of the payment mechanisms within basic medical insurance.

On the demand side, the basic medical insurance system should improve differentiated payment structures, prompting urban and rural residents to proactively embrace hierarchical diagnosis and treatment[6,7]. Encouraging the public to engage in graded diagnosis and treatment through the implementation of varied reimbursement policies in medical facilities at different levels has become a widely adopted strategy across most regions of the country[8].

However, it is worth noting that this approach often relies on economic incentives and tends to overlook both the psychological aspects of healthcare decision-making among individuals and the phenomenon of tertiary hospitals disproportionately attracting patients. The profit-driven motives of medical institutions and the limited capacity of primary healthcare facilities pose significant challenges in realizing the vision of comprehensive hierarchical diagnosis and treatment[9].

Research conducted by Shen Shuguang and Zhang Bo in the Pearl River Delta region reveals that residents' inclination to seek medical care at the primary level is notably low[8]. This is primarily attributed to the insufficient capacity and resources of primary medical facilities, which, in turn, erode trust in the concept of "primary first diagnosis." Further investigations have unveiled several underlying issues. Policy-driven primary medical institutions prioritize public health over medical care, and this is coupled with a lack of robust performance incentives, an incomplete compensation structure, antiquated salary and management systems within these primary healthcare facilities. These factors collectively contribute to the diminished motivation among primary healthcare staff[10-12]. Therefore, the establishment of a hierarchical diagnosis and treatment system faces significant hurdles, with the root cause residing in the inadequacies of medical care, rather than medical insurance[13].

In the 1990s, the United States witnessed the emergence of the concept and practice of "integrated healthcare" on the supply side of the medical field. This approach involved the consolidation of various healthcare service providers into a coordinated network, with the aim of delivering comprehensive, end-to-end healthcare services to patients, encompassing prevention, diagnosis, treatment, and rehabilitation[14]. Several studies have demonstrated the

effectiveness of Accountable Care Organizations (ACOs), a form of American medical consortium, in proactively controlling medical costs without compromising the quality of patient care[15-16]. Medicare in the United States has augmented incentives to control costs by innovating the payment model for medical consortia, thereby encouraging cost-effective healthcare delivery[17].

In recent years, the medical commonwealth model has emerged as a prominent representative of the reform strategies adopted by public hospitals. It is widely regarded by a majority of scholars as "the strategic choice for China's healthcare reform" and a pivotal measure in the ongoing and comprehensive supply-side structural transformation of China's healthcare system[18,19].However, in the early stages of its implementation in China, the member institutions within the medical commonwealth were integrated mainly in form, resulting in a dispersion of actual power, responsibility, and benefits. The lead hospitals often lacked effective incentive mechanisms, leading to reduced operational efficiency[20].

From a localized perspective, the county medical community stands out as the primary model for medical commonwealth construction at present. Its feasibility and practicality make it a significant breakthrough in the pursuit of an efficient and high-quality medical and healthcare service system. It bears practical significance in addressing the fragmentation within the healthcare service system and enhancing the overall efficiency of county-level medical and healthcare resources.As a result, the current implementation of the medical community model has evolved over time. It has transitioned from a diverse array of pilot projects at the outset to exhibit the characteristics of a more cohesive and compact medical community model[21].

The medical community introduces a multi-level principal-agent relationship involving "patient - medical insurance - hospital - doctor." However, the establishment of a tightly-knit medical community addresses the challenges posed by this complex relationship through a process of "integration." This integration aligns the interests of all parties within the medical system, transforming them into a "community of interests" that collectively serves the overarching objective of the entire system: maintaining the health of the entire population at the lowest possible cost[22,23].

Building upon this foundation, policy entrepreneurs in the early stages of the "bottom-up" development of the medical community collaborate with internal and external mechanisms once the "top-down" policy is later implemented. By reforming the medical insurance payment system, personnel performance evaluation and establishment mechanisms, family doctor sign-up procedures, and information sharing protocols, a new incentive compatibility mechanism is constructed to align the interests of government, hospitals, doctors, and patients.As a result, this restructuring leads to Pareto improvements and enhanced healthcare outcomes, ultimately facilitating the establishment of an effective hierarchical diagnosis and treatment system[2,24].

The existing literature primarily focuses on assessing the operational outcomes of local medical communities, with evaluations centered on the hypothesis that medical communities promote hierarchical diagnosis and treatment and optimize the allocation of medical resources, ultimately reducing the economic burden on patients and the medical insurance fund.

Firstly, the establishment of medical communities serves to enhance the allocation of healthcare resources by fostering two-way referral channels, strengthening the capacity of primary medical institutions, instilling trust in these institutions among patients, and establishing a positive feedback loop[25]. When it comes to hospitals, studies indicate that the implementation of medical communities in certain cities has enhanced the medical service capabilities of member institutions, bolstered overall competitiveness, improved medical quality, and facilitated two-way referral services[26]. It has been verified that the construction of a medical community in a sub-provincial city contributes significantly to hierarchical diagnosis and treatment, with secondary hospitals benefiting the most[27].

In the case of primary medical institutions, the example of Zhongxian County in Chongqing, which introduced the concept of a "vertical and horizontal union" medical community, offers a notable illustration. This reform led to increased business capacity of primary medical institutions by 25%, a 38% rise in the number of licensed physicians, an 84% increase in the average salary of medical staff, a 3.3 percentage point rise in the county-level consultation rate, and an 8.5 percentage point increase in the first-visit rate[28]. Empirical analyses conducted by Wang Shuyun and Sun Zesheng on the hierarchical diagnosis and treatment reform implemented in Shanghai in 2015, featuring family doctor contracts and unstructured medical unions, revealed a significant impact of the diagnosis and treatment capabilities and the treatment environment of primary medical institutions on patient satisfaction, consequently enhancing patient loyalty[29].

The medical community model in Tianchang City has achieved a comprehensive medical service governance mechanism that involves "hospitals, patients, doctors, and medical insurance." It has also laid the groundwork for a rural healthcare system centered on residents' health, characterized by the ability to receive initial diagnoses within 15 minutes and access specialized care within 50 kilometers[30].

A five-year reform initiative in the construction of a county medical community in Nagqu City, Tibet Province, has significantly increased the availability of high-quality medical resources, creating a three-tier county-level medical service system that connects counties and villages. This has led to a 53% reduction in referrals outside the county, essentially realizing the goals of keeping minor ailments at the township level and managing moderate diseases at the county level[31].

In terms of alleviating the financial burden on patients and optimizing the medical insurance fund, a case analysis of Yunxian County in Yunnan Province conducted by Zhu Jingmin and Duan Hui has yielded impressive outcomes[32]. The Yunxian medical community reform not only established a hierarchical diagnosis and treatment pattern but also significantly reduced the average medical treatment costs for residents while enhancing the efficiency of the medical insurance fund. In 2020, the balance of the medical insurance fund in Yun County reached nearly 20 million yuan, a substantial improvement compared to the overall city budget, which was incurring a deficit of approximately 1 million yuan.

Furthermore, Liao Dongping and colleagues leveraged the operating data from 12 compact medical communities in Sanming City, which implemented total medical insurance fund underwriting combined with C-DRG (Case-Based Payment System) collection and

payment[33]. Their extensive evaluation of the impact of medical insurance package payment reform on the operation and management of compact medical communities demonstrated that under this reform, medical communities can alleviate the financial burden on patients, increase hospital staff salaries, and bolster the balance of the medical insurance fund.

However, it's important to acknowledge that there remain several challenges in the local implementation of medical communities. Firstly, the top-level design of policies often lacks clear objectives and comprehensive plans, with insufficient systematic support from related policies. This frequently results in a situation where policies are symbolically implemented but lack substantial impact[34-35].

Secondly, the process of organizational integration sometimes fails in terms of resource allocation. Weaker grassroots institutions may find their resources further concentrated in more robust superior institutions, exacerbating the imbalance in healthcare provision, with the strong becoming stronger and the weak becoming weaker[9].

Additionally, the lack of effective incentive-compatible design hampers the motivation of partners to participate in these alliances and share resources. This can result in a distortion where the nominal collaboration actually fosters competition[36,37]. While some studies have shown that medical union reforms can enhance the efficiency of community medical and healthcare services to some extent, the implementation of these policies often increases the number of patients seeking care at community facilities without significant improvements in service quality or patient satisfaction[38].

At the present stage, system design, benefit distribution, information sharing, common resource management, and supervision methods all exhibit deficiencies. These shortcomings lead to high transaction costs in aspects like incentive management, coordination, information sharing, decision-making, and oversight. Addressing these challenges is crucial for the effective and efficient functioning of medical communities[39].

Based on a field investigation of 11 pilot counties involved in the construction of medical communities in Zhejiang Province, Xu Yuyun and Yu Jianxing identified the reform of the medical insurance payment system as a critical factor influencing the success or failure of medical community construction[40]. In the Zhejiang medical community pilot program, medical insurance payments continue to be primarily based on a post-payment system controlled by total expenditure. Project-based payments still dominate, with incremental budgeting predominantly determining the total budget base. The incentive mechanisms related to sharing overspending and retaining surpluses need improvement, significantly constraining the realization of the policy objectives of the medical community.

Wu Yixin and Li Yueping found that the implementation of medical insurance package payment in a tightly-knit county medical community in Fujian encouraged patients to seek treatment within the county but did not significantly promote patient triage to grassroots-level healthcare facilities[41]. In practice, the impact of medical insurance balances on motivating collective action is relatively weak, and the effectiveness of supporting policy tools such as medical insurance payment rates and salary structures in aligning individual behavior with collective goals is limited[42].

The total prepayment system has played a positive role in controlling medical expenses

within county medical communities and preventing excessive healthcare behaviors. However, due to the absence of internal incentive and restraint mechanisms, member institutions may still decline patients, encourage patient transfers to other hospitals, prefer non-medical insurance patients, and avoid the use of expensive medications and high-value consumables, actions that are inconsistent with their intended roles and functions[43].

Most of the existing literature is focused on describing the policies and the current state of medical union and medical community construction. Early studies primarily involved evaluating the effects through investigations of individual urban medical community pilot programs, with limited use of empirical testing.

The theoretical significance of this paper lies in its effort to rationalize the incentive compatibility mechanism among participants in various systems created by the reform of package payments for medical insurance in Sichuan Province and the internal reform of medical communities. It contributes to enriching the theory related to hierarchical diagnosis and treatment in China.

The practical significance of this paper is that it employs a rigorous approach, combining sample selection with the differential difference (PSM-DID) method and reliable data to investigate the impact of the pilot compact medical community in 37 districts and counties of Sichuan Province on patients' choices for medical treatment, hospital diagnosis and treatment behaviors, and medical resource allocation. This research offers valuable insights on how to further enhance the construction of compact medical communities and promote the hierarchical diagnosis and treatment system in China.

3.Theoretical mechanism analysis and research hypothesis

The concept of "incentive compatibility" was initially introduced by Hurwicz[44]. In economic activities, when there is an institutional arrangement that enables individuals to align their pursuit of self-interest with the objective of maximizing collective value, it is referred to as "incentive compatibility." This theory was first applied by Mauris to address principal-agent problems[45].

The incentive compatibility framework for the compact medical community in Sichuan Province, aimed at achieving graded diagnosis and treatment under the medical insurance package payment system, is depicted in Figure 1. This framework comprises four institutional actors: government departments, the medical community, doctors, and patients, and three principal-agent relationships, which are patient-government departments, government-medical community, and medical community-doctor relationships.

The Implementation Plan strives to effectively align the interests of principals and agents at various levels of the principal-agent chain through a range of policy measures and system optimizations. The objective is to motivate each institutional actor to pursue their self-interest while simultaneously advancing the collective goal of maximizing overall value.

The construction of a tightly-knit medical community at the county level in Sichuan

Province involves four institutional subjects and three levels of principal-agent relationships. In these relationships, there is a significant information asymmetry and misalignment of objective functions between principals and agents within the traditional non-medical community model. Additionally, the government departments, the medical community, and the medical practitioners exist at different levels or within different departments, making the internal and external relationships of the medical community complex.

The establishment of a tight medical community in Sichuan Province relies on various factors, including the reform of the medical insurance payment method, enhancements to the employment mechanism, adjustments to the salary incentive mechanism, improvements in the family doctor signing system, modifications to medical service pricing, the comprehensive allocation of resources, the creation of smooth two-way referral pathways, the promotion of the overall integration of medical and preventive services, the enhancement of information systems, and the implementation of financial investments and supervisory responsibilities. These efforts collectively contribute to the creation of an incentive compatibility mechanism for hierarchical diagnosis and treatment.

The basic medical insurance system is the largest financial contributor to medical institutions. A well-designed medical insurance system can effectively influence and regulate patients' healthcare-seeking behaviors[46], as well as motivate doctors at various levels of healthcare facilities[47], including grassroots hospitals, to actively participate. This is conducive to achieving the reform objectives of graded diagnosis and treatment.

In the realm of medical insurance system design, payment system reform is pivotal to the success or failure of medical community construction[40]. In Sichuan Province's "implementation plan," key proposals include:Enhancing the total budget management of the medical insurance fund;Establishing the mechanism of "surplus retention" and "reasonable overspending sharing."Adjusting the total control indicators to be more favorable to grassroots medical and healthcare institutions;Allowing medical communities that have implemented financial, information, and settlement integration to implement a "unified total amount" for medical insurance and adjust the total amount index within the medical community;Implementing a comprehensive "multi-compound medical insurance payment method based on disease type."These measures collectively address the core aspects of the medical insurance system and its payment mechanisms, contributing to the promotion of graded diagnosis and treatment reform.

In terms of asset management, the medical community is authorized to coordinate the deployment of medical equipment and facilities in accordance with relevant regulations on asset disposal. Concerning informatization, the policy promotes the integration of information systems across all member units of the medical community, facilitating data sharing and medical insurance settlement integration, which creates the conditions for the realization of total medical insurance advancement. These efforts aim to enhance the digital operations of the medical community, such as resource allocation, business operations, and efficiency monitoring. They also encourage the continuous recording of electronic medical records and electronic health records.

The medical insurance package payment policy, combined with the construction of the

medical community, encourages a shift from focusing solely on "disease" to a broader focus on "health." The delegation of asset management authority and the advancement of informatization levels incentivize the medical community to efficiently share and allocate medical resources between primary and higher-level medical institutions. This leads to cost reduction, improved medical service effectiveness, and the attainment of medical insurance balance[48].

Doctors play a central role in providing medical services and are key to achieving the goals of all stakeholders in the compact county medical community. The "Implementation Plan" grants the medical community full autonomy in internal management, giving them the authority to manage staffing and salary distribution. It encourages the establishment of a flexible employment mechanism that allows for adjustments in staffing levels, facilitates job rotation, and promotes two-way personnel flow within the medical community. Additionally, the plan promotes the adoption of a contract service fee for family doctors, shifting the focus of the medical community from treating pre-existing diseases to giving equal attention to both pre-existing and non-existing diseases. It also suggests a salary distribution mechanism that prioritizes clinical front-line and grassroots medical staff, with reasonable wage gaps.

These measures, such as the flexible employment mechanism, family doctor contract service fees, and the distribution of salaries favoring grassroots medical staff, encourage doctors from higher-level hospitals to work at grassroots levels and motivate grassroots doctors. This promotes the realization of "two-way referral" and the integration of medical and preventive services within the community[2].

Under the construction of the medical community, there has been an improvement in the medical service capacity of grassroots medical institutions. The "Implementation Plan" further recommends enhancing differentiated medical insurance payment policies to guide insured individuals to prioritize their initial diagnosis at the grassroots level. For referred hospitalized patients who meet the criteria, continuous calculations of the starting payment line are suggested. Moreover, the key component of the medical community's construction, the family doctor contract system[2], along with differentiated medical insurance payment policies, encourages insured patients to actively seek initial diagnoses at the grassroots level[49-50].

Regarding financial input, the plan states that subsidy funds for member units of the medical community should be allocated according to the community's construction and development needs. Additionally, it suggests that basic public health service funds should be managed and used by the medical community as a whole, following the total budget of the permanent population served by the community. This approach ensures a stable source of funding for the medical community's development and construction while promoting the integration of medical service provision and public health service provision.

Local government departments also bear the responsibility of improving the pricing of medical services, supervising the quality of healthcare, and enhancing the assessment and evaluation system for the construction of the medical community. These policies collectively provide strong support for the construction of the medical community, ensuring the people's health is safeguarded, the sustainability of the medical insurance fund, efficient financial

subsidies, and effective public health investment. Ultimately, these measures aim to achieve the goal of hierarchical diagnosis and treatment.

The "Implementation Plan" has established specific work goals for the construction of the medical community, with a focus on increasing consultation rates and enhancing the role of grassroots medical institutions in providing healthcare. These goals include striving for a 90% consultation rate at the county level and increasing the proportion of outpatient, emergency, and inpatient visits at grassroots medical institutions.To evaluate the effectiveness of these goals and the incentive compatibility mechanism framework, several hypotheses are proposed:

H1: The county-level consultation rate of urban and rural residents and urban employee patients in the pilot districts of the medical community increased, and more people proactively sought initial diagnoses at grassroots medical institutions.

H2: The medical service capacity of the pilot districts and counties of the medical community has improved, with medical institutions at all levels effectively fulfilling their functional roles. This would result in the medical treatment pattern of "major diseases go to the hospital, minor diseases go to the community."

H3: Medical institutions at all levels within the medical community in the pilot districts and counties have successfully optimized and allocated resources more reasonably.

These hypotheses will be tested to determine the impact of the medical community's construction on the healthcare system and the achievement of hierarchical diagnosis and treatment.

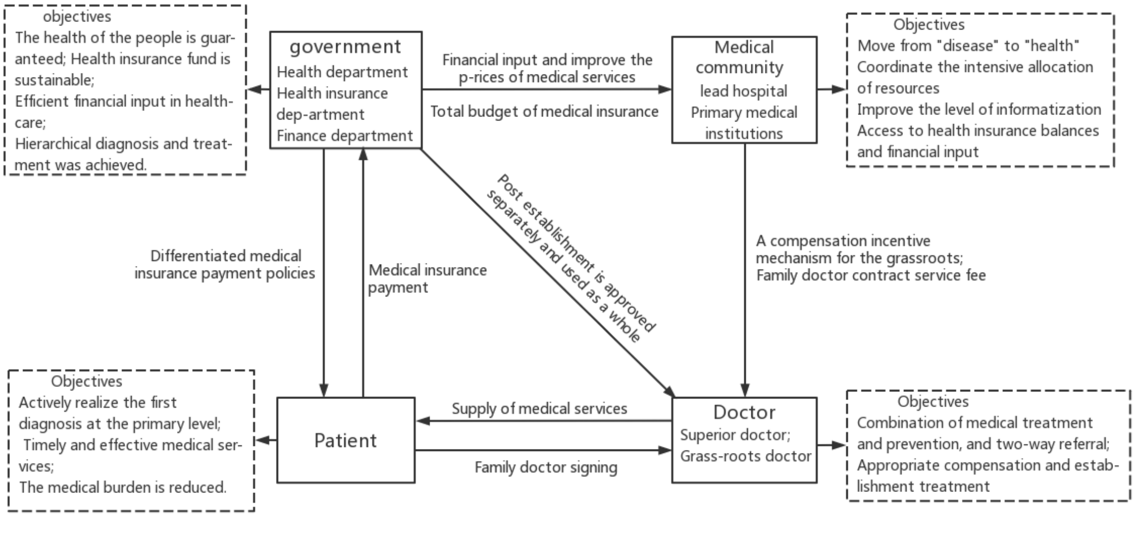

FIG. 1 Incentive compatibility framework of medical community to realize hierarchical diagnosis and treatment under "package payment" of Sichuan medical insurance

4.Data, variables and model

4.1. Data source

The data sources used in this paper are essential for conducting empirical research on the effects of pilot compact county medical communities in Sichuan Province. The main data sources are as follows:

Sichuan Provincial Health Statistical Yearbook: This publication is a critical source for collecting data related to healthcare services, infrastructure, and various health-related indicators. It provides a comprehensive overview of the healthcare system in Sichuan Province and contains historical data from 2008 to 2021.

Wind Database: The Wind database is a financial and economic database that contains a wide range of financial and economic data for Chinese provinces and regions. In this paper, it was used to obtain various control variables that could potentially impact the performance of the pilot compact county medical communities.

"Implementation Plan for the Construction of Pilot Medical Community in Sichuan Province": This official document is used to identify which counties in Sichuan Province were designated as pilot areas for the construction of compact county medical communities.Table 1 serves as a reference for the selection of pilot counties.

The data from these sources are crucial for conducting a comprehensive and data-driven analysis of the impact of the pilot compact county medical communities. Researchers can use this data to examine various variables and indicators that are relevant to the study's objectives and hypotheses.

Table 1 List of pilot counties for the construction of tight county medical community in Sichuan Province

City	Number	List of counties (city,districts)	City	Number	List of counties (city,districts)
Chengdu	5	Xindu , Qingbaijiang, Qionglai , Xinjin and Pujiang	Yibin	3	Xingwen ,Jiang 'an ,Junlian
Zigong	2	Gongjing , Ziliujing	Guang'an	2	Guang 'an , Huaying
Panzhihua	2	Miyi , Yanbian	Dazhou	1	Tongchuan
Luzhou	2	Lu , Hejiang	Bazhong	1	Pingchang
Deyang	4	Mianzhu , Guanghan ,Zhongjiang and Luojiang	Ya'an	1	Shimian
Mianyang	1	Pingwu	Meishan	1	Qingshen
Guangyuan	1	Chaotian	Ziyang	1	Anyue
Suining	1	Anju	Aba	2	Wenchuan,Jiuzhaigou
Neijiang	1	Longchang	Garzi	2	Ganzi, Seda
Le shan	1	Muchuan	Liangsha	2	Dechang and Xichang
Nanchong	1	Shunqing	Total	37	

4.2.Variable Settings

The paper's research involved a range of explanatory variables, dependent variables, and control variables. These variables are essential for analyzing the impact of pilot compact county medical communities in Sichuan Province. It is important to note that data availability and consistency are crucial for conducting robust empirical research.

Here are some key points regarding variable settings in the paper:

Explanatory Variables: These are variables that are used to explain or predict changes in the dependent variables (the outcomes). In the context of the paper, the primary explanatory variable of interest is whether a county was part of the pilot compact county medical

community program or not.

Dependent Variables: These are the outcomes or variables that the paper aims to study and analyze. The paper looks at various dependent variables, such as the county-level consultation rate, medical service capacity, and resource allocation. The goal is to assess how these variables changed in pilot areas compared to non-pilot areas.

Control Variables: Control variables are used to account for potential confounding factors or other variables that could affect the outcomes. They help ensure that any observed effects are not due to other factors. The paper used various control variables obtained from the Wind Database.

The paper acknowledges inconsistencies in the subjects of the indicators used. For instance, the Yearbook reported data on consultations for various types of medical institutions, but data on health personnel were available for a specific subset of institutions. To address this, the paper analyzed and tested the effects based on the available data in a manner that is consistent with the subject matter of each indicator.

This comprehensive approach to variable selection allows the paper to conduct a thorough analysis of the impact of the pilot compact county medical communities while considering potential confounding factors.

Table 2 Variables and index systems of promoting hierarchical diagnosis and treatment by county medical communities

Variables	指标		释义
Explanatory Variables	Medical community pilot system or not(ifygt)		0-1 variables, ifygt = 1, indicates the medical community pilot; ifygt = 0 indicates no medical community pilot.
Dependent Variables	Residents' choice of medical treatment	Per capita diagnosis and treatment(*dtp*)	The per capita number of visits in medical and health institutions indicates the frequency of residents going to medical and health institutions；The per capita number of visits to the public hospitals indicates the frequency of residents going to high-level medical institutions;The per capita number of visits in primary medical and health institutions is the same.
		The proportion of diagnosis and treatment(*dtr*)	The construction of the proportion of public hospitals and the proportion of primary medical and health institutions indicates the tendency of people to choose medical treatment to go to different medical institutions.
	Diagnosis and treatment behavior choice	People in hospital per capita(*htp*)	The per capita hospitalization of medical and health institutions represents the frequency of hospitalization in all medical institutions; the per capita resident of public hospital indicates the frequency of hospitalization in public hospital;The average hospitalization number of permanent residents in primary medical and health institutions is the same.
		The proportion of inpatients(*htr*)	The proportion of inpatients in public hospitals and the inpatients in primary medical and health institutions indicates the tendency of county people to be hospitalized in different medical institutions.
		Surgery rate in hospitalized patients(*htsr*)	The operation rate of inpatients in hospitals and public hospitals is used to indicate the behavior tendency of hospital diagnosis and treatment behavior.
	Allocation of medical resources	Health workers per thousand people(*pher*)	The number of health personnel per 1,000 permanent residents in hospitals, health personnel per 1,000 rural residents in township health centers, and health personnel per 1,000 urban residents in community health service centers represents the allocation of human resources at various levels of medical institutions.

	Number of beds per thousand people(*phbed*)	The number of beds per 1,000 permanent residents of medical institutions, primary medical and health institutions, community health service centers, township health centers and outpatient departments indicates the allocation of medical facilities by medical institutions at all levels.
控制变量	The level of population aging（*agingdegree*）	The proportion of the population over 65 years old in the total population is expressed in units:%
	sex structure （*sexrate*）	The ratio of male to female population represents the local gender structure, unit (%)
	Urbanization level（*urpopustr*）	The ratio of the urban population to the total permanent resident population indicates the local urbanization level, per unit:%
	The number of confirmed COVID-19 cases（*covid_19*）	The number of COVID-19 cases indicates the impact of COVID-19 from the end of 2019 to 2021, unit: person
	per capita gross domestic product（*lngdp*）	Per capita GDP is log taken to indicate the level of local economic development

4.3. Descriptive Statistical Analysis

The descriptive statistical analysis presented in Table 3 compares key indicators related to the hierarchical diagnosis and treatment promoted by the construction of the county medical community. The analysis includes both the experimental group, which consists of counties participating in the medical community pilot, and the control group, which comprises counties that did not participate in the pilot.

Key findings from the descriptive analysis include significant differences in various healthcare-related indicators between the two groups. These indicators include:Per Capita Outpatient Visits,The number of outpatient visits per person in healthcare institutions at different levels;Per Capita Inpatient Visits,The number of inpatient visits per person in healthcare institutions at different levels;Inpatient Surgical Rate,The proportion of inpatients undergoing surgery;Healthcare Personnel per Thousand People,The number of healthcare personnel per thousand people in the population;Hospital Beds per Thousand People,The number of hospital beds per thousand people in the population.

These significant differences suggest the possibility of selection bias in the determination of pilot areas. In other words, the areas selected for the medical community pilot may have distinctive characteristics. This could lead to endogeneity issues if straightforward Ordinary Least Squares (OLS) estimation is conducted. To address this, it is necessary to first tackle the problem of sample selection bias before evaluating the effects of the medical community pilot.

Table 3 Descriptive statistical analysis of the pilot promotion of hierarchical diagnosis and treatment in county medical communities

变量	Medical institution level	Experimental group			Control group		
		sample capacity	average value	standard deviation	sample capacity	average value	standard deviation
dtp	Medical and health institutions	333	7.348	11.666	1313	5.627	9.952
	Public hospitals	332	2.029	2.621	1309	1.677	1.904
	Primary medical institutions	333	5.020	9.601	1313	3.665	8.628
htp	Medical and health institutions	333	0.254	0.411	1313	0.197	0.265
	Public hospitals	332	0.135	0.205	1309	0.106	0.131
	Primary medical institutions	333	0.088	0.209	1313	0.063	0.137
htsr	Hospitals	444	0.239	0.132	1746	0.225	0.126
	Public hospitals	443	0.254	0.149	1738	0.235	0.131

	Hospitals	333	517.789	750.142	1314	448.455	527.020
pher	Health clinics in towns and townships	481	187.785	86.813	1823	198.775	97.419
	Community health service centers	396	63.884	55.230	1357	64.848	61.783
	Public hospitals	333	371.538	565.627	1314	305.853	329.266
phbed	Community health service station	333	3.465	22.780	1314	1.508	9.185
	Outpatient department	333	1.105	7.145	1314	0.332	2.395
	agingdegree	259	14.201	3.633	1022	13.416	3.862
	sexrate	481	1.046	0.048	1887	1.051	0.043
	urpopustr	333	46.201	15.092	1314	44.203	18.918
	covid_19	259	20.174	88.084	1022	16.460	77.407
	lngdp	481	10.408	0.616	1889	10.242	0.658

4.4.Models

The paper employs the PSM-DID model, which combines the Propensity Score Matching (PSM) method and the Double Difference (DID) method. This approach effectively addresses the presence of observable and unobservable confounding factors between the treatment group (medical community pilot areas) and the control group.In the context of the compact county medical community pilot policy, it essentially represents a non-randomized experiment (or quasi-natural experiment). Therefore, conducting a policy evaluation directly through the DID approach can be susceptible to self-selection bias, potentially leading to endogeneity. However, using the PSM method enables the matching of specific control group samples to each treatment group sample. This makes the quasi-natural experiment approximation more like a random one, reducing the impact of self-selection bias and increasing the reliability of the analysis.In essence, PSM-DID is a rigorous approach to evaluate the impact of policies or interventions in a non-randomized setting while minimizing potential biases. It allows for more robust and unbiased assessments of policy effectiveness[51,52].

4.4.1. Propensity score matching (PSM)

Propensity Score Matching (PSM) is a statistical method used to create a counterfactual framework that helps mitigate the impact of selection bias when estimating parameters for districts and counties that share similar characteristics and have implemented the county-level compact health community pilot project. The form of the binary logit model for estimating the propensity score is as follows:

$$\ln\left(\frac{p_i}{1-p_i}\right) = \beta_0 + \beta_1 x_i' + u_i \qquad (1)$$

Where:$p_i = p(X_i) = Pr(T_i = 1|X_i)$represents the propensity score of the sample counties, while x_i'signifies the covariates selected through iterative comparison, including population aging level, gender structure, urbanization level, the number of COVID-19 cases, per capita gross domestic product, and resource allocation of medical and health institutions (health personnel per thousand residents). In this study, caliper matching with a radius of 0.05 was employed to retain matched samples while eliminating unmatched ones.

4.4.2.Double checking (DID)

To further control for potential unobservable variables, this paper incorporates individual and time-fixed effects. Using panel data, the influence of county-level compact medical community construction on residents' choice of medical treatment can be explored using the following formulas:

$$y_{it}^{1s} = \beta \times c_i + \theta_i + x_{it}'\gamma + \tau_t + \epsilon_{it} \qquad (2)$$

y_{it}^{1s} represents the per capita visits to s medical institutions at time t. c_i is a binary variable, taking the value 1 when the district and county undergo the compact county medical community reform, and 0 otherwise. θ_i stands for the fixed effect of the district and county, controlling for heterogeneity at the district and county level. x_{it}' represents a set of control variables including the level of population aging, gender ratio urbanization level, COVID-19 cases, per capita gross domestic product, and resource allocation of medical and health institutions (health personnel per 1,000 permanent population). τ_t represents the fixed effect of the year, controlling for the common time impact of cities and states in different periods. ϵ_{it} is the residual, and robust standard error is employed for estimation.

$$y_{it}^{2s} or y_{it}^{3s} = \beta \times c_i + \theta_i + x_{it}'\gamma + \tau_t + \epsilon_{it} \quad (3)$$

y_{it}^{2s} represents the per capita admissions of prefecture-prefecture *i* in *s* medical institutions with time *t*, and y_{it}^{5s} represents the rate of inpatient surgery in county *i* in *s* medical institutions at time *t*. Other indicators are the same as formula (2).

Finally, how and the construction of medical community affects the resource allocation of medical institutions. The formula is as follows:

$$y_{it}^{4s} or y_{it}^{5s} = \beta \times c_i + \theta_i + x_{it}'\gamma + \tau_t + \epsilon_{it} \quad (4)$$

y_{it}^{4s} represents the health personnel per thousand permanent population in s medical institutions at time *t*. y_{it}^{5s} represents the number of beds per thousand permanent resident population in s medical institutions at time *t*. Other indicators are the same as formula (2).

5. Empirical regression results

5.1. Main effect:

5.1.1 diversion effect

Table 4 illustrates the impact of the intensive medical community in Sichuan Province under the package payment of medical insurance on guiding patients towards medical shunting. In general, the per capita number of medical and health institutions in the pilot districts of the medical community increased significantly, by 0.434 times. When looking at medical institutions of different levels, it is observed that the per capita visits to public hospitals in the pilot areas and counties did not change significantly. However, the per capita visits to basic medical institutions increased significantly, by 0.340 times.

While it might appear that there hasn't been a significant change in the proportion of consultations between public hospitals and primary medical institutions, it is important to note that this balance has attracted the return of severe patients and encouraged medical shunting at the grassroots level. This effect seems to have somewhat canceled each other out in the

structure. The results suggest that the county-level consultation rate has improved following the pilot of the medical community, and the pilot project has indeed played a role in guiding county patients to seek medical care locally and promoting initial diagnoses at the grassroots level to a certain extent.

Table 4 Influence of county health community construction on patients' medical shunt

Explanatory variable	Dependent Variables				
	dtp			*dtr*	
	Medical and health institutions	Public hospitals	Primary medical institutions	Public hospitals	Primary medical institutions
did	0.434**	0.085	0.340**	0.004	-0.004
	(0.185)	(0.088)	(0.134)	(0.011)	(0.011)
Constant	-5.559	-2.320	-2.714	0.468	0.501*
	(4.971)	(2.222)	(3.674)	(0.318)	(0.293)
Annual effect	yes	yes	yes	yes	yes
Individual effect	yes	yes	yes	yes	yes
R-squared	0.901	0.949	0.883	0.945	0.951
Sample capacity	390	387	390	390	387

Robust standard errors in parentheses, *** p<0.01, ** p<0.05, * p<0.1

5.1.2.Diagnosis and treatment effect

Table 5 provides insights into the impact of the county-level compact medical community in Sichuan Province on guiding the treatment behavior of medical institutions at all levels under the package payment of medical insurance. Notably, the total per capita inpatient visits to medical and health institutions saw a significant increase of 0.02[illegible]

When examining per capita inpatient visits to public hospitals and grassroots medical institutions, it becomes evident that the pilot of the medical community mainly led to an increase in the per capita inpatient visits to public hospitals. However, the proportion of inpatient visits in public hospitals and grassroots medical institutions in the pilot districts and counties did not undergo significant changes, which is also reflected in the number of inpatient visits.

Furthermore, the total inpatient operation rate of hospitals and the inpatient operation rate of public hospitals increased by 4.4% and 5%, respectively. This indicates that the medical community pilot has not only encouraged initial diagnoses at the grassroots level but has also enhanced the medical service capabilities of county-level medical and health institutions. It has also promoted public hospitals to resume their role in dealing primarily with complex and severe diseases, thereby further optimizing the healthcare pattern of "major diseases being addressed in hospitals, and minor diseases being managed in the community."

Table 5 Influence of county medical community construction on diagnosis and treatment behavior of medical institutions at all levels

Explanatory variable	Dependent Variables						
	htp			*htr*		*htsr*	
	Medical and health institutions	Public hospitals	Primary medical institutions	Public hospitals	Primary medical institutions	Hospitals	Public hospitals
did	0.020***	0.011***	0.009***	0.007	0.006	0.044**	0.050**
	(0.007)	(0.004)	(0.003)	(0.009)	(0.013)	(0.020)	(0.023)
Constant	-0.329*	-0.067	-0.176**	-0.288	1.302***	2.411***	2.651***

	(0.188)	(0.107)	(0.088)	(0.249)	(0.327)	(0.667)	(0.790)
Annual effect	yes	yes	yes	yes	yes	yes	yes
Individual effect	yes	yes	yes	yes	yes	yes	yes
R-squared	0.922	0.955	0.916	0.969	0.964	0.708	0.664
Sample capacity	390	387	390	390	387	390	387

Robust standard errors in parentheses, *** p<0.01, ** p<0.05, * p<0.1

5.1.3.Resource allocation effect

Table 6 illustrates the impact of the county-level compact medical community in Sichuan province on guiding the behavior of medical institutions at all levels in diagnosis and treatment under the package payment method of medical insurance. Several key observations can be made from the data:There was no significant change in the number of health personnel per 1,000 population in medical institutions at all levels. This suggests that the pilot medical community has not significantly affected the allocation of health human resources.The number of beds per 1,000 population in community health service stations increased significantly by 1.678. This indicates that the pilot program has led to an increase in the allocation of medical facility resources at the community level.Conversely, the number of beds per 1,000 population in outpatient departments of hospitals decreased significantly by 0.739. This decrease may reflect a shift in resource allocation, potentially indicating that medical resources are being redirected away from hospital outpatient departments.In summary, the pilot medical community program has had a mixed impact on the allocation of resources, with health personnel remaining largely unchanged, but with notable changes in the allocation of medical facility resources.

Table 6 Influence of county medical community construction on the allocation of medical resources

Explanatory variable	Dependent Variables							
	pher			*phbed*				
	Hospitals	Health clinics in towns and townships	Community health service centers	Public hospitals	Community health service centers	Community health service station	Health clinics in towns and townships	Outpatient department
did	-15.665	-18.045	-1.471	-3.363	0.769	1.678**	0.785	-0.739*
	(14.558)	(11.964)	(5.976)	(13.646)	(1.958)	(0.760)	(9.914)	(0.436)
Constant	-721.077**	730.793	105.614	-56.426	21.515	-1.753	-223.346	19.425**
	(354.627)	(473.758)	(177.488)	(284.466)	(32.632)	(22.232)	(181.756)	(9.335)
Annual effect	yes	yes	yes	yes	yes	yes	yes	yes
Individual effect	yes	yes	yes	yes	yes	yes	yes	yes
R-squared	0.965	0.901	0.851	0.928	0.729	0.834	0.770	0.475
Sample capacity	392	360	304	392	392	392	392	392

Robust standard errors in parentheses，*** p<0.01, ** p<0.05, * p<0.1

5.2.Parallel trend test

The DID model relies on the parallel trend hypothesis, which assumes that, before implementing the policy, cities that implement outpatient coordination and those that do not should exhibit parallel trends in various factors, including the number of medical institutions at all levels, the outpatient and emergency hospitalization rates of public hospitals, and the allocation of medical resources in public hospitals. To test this hypothesis, the paper employs the event study method proposed by Jacobson et al.[53] for a parallel trend analysis.

The analysis focuses on the main regression results related to the three effects mentioned above. It compares the data in the pre-implementation phase (pre3) to the data in the first three phases of the pilot program. The results of the parallel trend test in Table 7 and Table 8 reveal that, except for the number of beds per 1,000 population in hospital outpatient departments, the coefficients of the variables analyzed in this paper are not statistically significant in the pre-implementation period (pre3, pre2). However, they become significant in the current and subsequent periods (current, post1, post2) following the implementation of the pilot policy.

In order to avoid issues related to multicollinearity, the baseline group was defined as the first period before the policy (pre1) and was excluded from the analysis. This suggests that, prior to the policy implementation, there were no significant differences in the number of outpatient visits, inpatient admissions, inpatient surgery rates, the number of health workers per 1,000 population, and the number of beds per 1,000 population at medical institutions of all levels in different cities and states implementing outpatient coordination at different times. This indicates that other factors did not significantly influence these factors. In essence, the study samples passed the parallel trend test.

Table 7 Parallel trend test of diversion effect and resource allocation effect

Explanatory variable	Dependent Variables			
	dtp		*phbed*	
	Medical and health institutions	Primary medical institutions	Community health service station	Outpatient department
pre_3	0.089	0.117	1.122	-0.739
	(0.359)	(0.284)	(1.154)	(0.601)
pre_2	-0.070	-0.065	0.961	-0.318
	(0.306)	(0.213)	(1.217)	(0.457)
current	0.220	0.214	3.473**	-0.915
	(0.284)	(0.198)	(1.441)	(0.646)
post_1	0.396	0.350*	1.541*	-0.954
	(0.290)	(0.211)	(0.914)	(0.708)
post_2	0.661**	0.456**	1.884*	-1.186
	(0.278)	(0.218)	(0.985)	(0.809)
Constant	-5.274	-2.485	0.038	19.330**
	(4.965)	(3.662)	(22.665)	(9.426)
Annual effect	yes	yes	yes	yes
Individual effect	yes	yes	yes	yes
R-squared	0.902	0.884	0.836	0.477
Sample capacity	390	390	392	392

Robust standard errors in parentheses，*** p<0.01, ** p<0.05, * p<0.1

Table 8 Parallel trend test of diagnosis and treatment effect

Explanatory variable	Dependent Variables				
	htp			*htsr*	
	Medical and health institutions	Public hospitals	Primary medical institutions	Hospitals	Public hospitals
pre_3	0.001	-0.000	-0.001	0.007	-0.008
	(0.011)	(0.007)	(0.004)	(0.023)	(0.027)
pre_2	-0.001	0.001	-0.002	0.009	-0.000
	(0.011)	(0.007)	(0.005)	(0.019)	(0.023)
current	0.014	0.008	0.008*	0.045	0.040
	(0.010)	(0.006)	(0.004)	(0.041)	(0.049)

post_1	0.020*	0.012*	0.007	0.046**	0.046*
	(0.011)	(0.007)	(0.004)	(0.021)	(0.024)
post_2	0.025**	0.013**	0.011*	0.053*	0.058*
	(0.011)	(0.006)	(0.006)	(0.028)	(0.034)
Constant	-0.322*	-0.065	-0.176**	2.421***	2.651***
	(0.190)	(0.108)	(0.088)	(0.660)	(0.782)
Annual effect	yes	yes	yes	yes	yes
Individual effect	yes	yes	yes	yes	yes
R-squared	0.923	0.956	0.916	0.709	0.664
Sample capacity	390	387	390	390	387

Robust standard errors in parentheses， *** p<0.01, ** p<0.05, * p<0.1

5.3.Placebo test

To further ensure the robustness of the results, and exclude the influence of other policy changes or random factors, this paper conducted a placebo test using a fictional policy time frame and a fictional treatment group. In this test, the integration of urban and rural residents' medical insurance in 2016 might affect the dependent variables of interest in this study. Following the approach of Liu Ruiming and Zhao Renjie[54], the pilot timeframe was moved two years ahead to 2017. The results showed that, with the exception of a few variables with weak significance, the estimated results of the dependent variables were not significant（S2_file）. Following the approach of Cai et al.[55], 37 districts and counties were randomly selected from the 183 sample districts and counties to serve as the fictitious treatment group cities. The remaining districts and counties were designated as the fictitious control group cities. This was done to obtain coefficient estimates for the impact of the county's compact medical community construction pilot on the public's medical treatment triage, hospital diagnosis and treatment behavior, and medical resource allocation.

The entire process was repeated 500 times, resulting in 500 sets of regression coefficients and their corresponding P-values. By visualizing the kernel density distribution and P-value distribution of these 500 coefficients, it becomes apparent that the regression coefficients are centered around zero and follow a normal distribution except for the number of beds in the community health service station. The majority of regression results are not statistically significant. The main effect regression results are positioned at the tail end of the positive distribution, representing rare occurrences.Therefore, it can be concluded that the main effect results presented in this paper are not due to confounding factors.The results displays the kernel density test graph for each variable related to the community construction pilot's impact on medical treatment triage, hospital diagnosis and treatment behavior, and medical resource allocation.

5.4.Robustness test

To ensure the robustness of the main regression results, a robustness test was performed by adjusting the data years. The analysis was conducted using data from 2016 to 2021. The estimated coefficients and their significance obtained from the results presented in Table 9 and Table 10 were found to be consistent with the original sample results. This indicates that the main regression results are robust and hold across different data years.

Table 9 Parallel trend test of diversion effect and resource allocation effect in the medical community pilot

Dependent Variables

Explanatory variable	*dtp*	
	Medical and health institutions	Primary medical institutions
did	0.335*	0.279*
	(0.199)	(0.148)
Constant	-5.426	-2.294
	(5.884)	(4.245)
Annual effect	yes	yes
Individual effect	yes	yes
R-squared	0.910	0.891
Sample capacity	321	321

Robust standard errors in parentheses，*** p<0.01, ** p<0.05, * p<0.1

Table 10 Parallel trend test of the diagnosis and treatment effect in the medical community trial

Explanatory variable	Dependent Variables				
	htp			*htsr*	
	Medical and health institutions	Public hospitals	Primary medical institutions	Hospitals	Public hospitals
did	0.017**	0.009*	0.009**	0.041**	0.051**
	(0.007)	(0.004)	(0.004)	(0.020)	(0.024)
Constant	-0.163	-0.059	-0.104	2.455***	2.786***
	(0.217)	(0.154)	(0.102)	(0.773)	(0.916)
Annual effect	yes	yes	yes	yes	yes
Individual effect	yes	yes	yes	yes	yes
R-squared	0.929	0.960	0.925	0.734	0.694
Sample capacity	321	321	321	321	319

Robust standard errors in parentheses，*** p<0.01, ** p<0.05, * p<0.1

6. Conclusions and policy recommendations

6.1. Conclusion

The conclusions drawn in this paper primarily focus on the impact of the pilot implementation of the compact county medical community in Sichuan Province on the hierarchical diagnosis and treatment under the package payment method of medical insurance. The main findings are as follows:The pilot of the county medical community significantly increased the county consultation rate among residents in the pilot areas, encouraging them to seek primary medical care and actively promoting first-level diagnosis and treatment.The county medical community pilot led to an improvement in the hospitalization rate of medical institutions at all levels in the pilot areas, particularly in public hospitals at the county level. Additionally, the pilot enhanced the inpatient operation rate in hospitals, indicating improved county-level medical service capacity and the redirection of hospitals to focus on treating severe and complex cases.The pilot county medical community failed to promote the sinking of medical infrastructure and human resources.These findings shed light on the effects of the compact county medical community pilot on healthcare delivery and resource allocation under the framework of medical insurance package payment.

6.2. Policy suggestions

6.2.1.Strengthening the Promotion of Medical Community Pilot Policies

This paper has demonstrated that the pilot implementation of the county medical

community in Sichuan Province has effectively guided county patients to seek medical treatment and encouraged first-level diagnosis at grassroots healthcare facilities. These outcomes represent crucial steps towards the gradual realization of hierarchical diagnosis and treatment within the medical community. They also provide the foundation for a positive feedback loop within the four-way incentive compatibility mechanism involving the government, medical community, physicians, and patients. To further enhance the success of the county medical system, it is imperative to bolster public awareness and understanding of the medical community pilot policies. Achieving this can be facilitated through differential medical insurance payment policies, the enhancement of the "family doctor" contract system, and the continuous improvement of medical service capacity within the medical community. These efforts will instill greater confidence among county patients in the county's healthcare system.

6.2.2. Enhancing Financial Subsidies for Primary Medical Resource Allocation

In terms of the number of beds per 1,000 people, only the number of beds in community health service stations has increased significantly after the medical community pilot but has not passed the placebo test, and there is no significant change in the bed allocation of other primary medical institutions such as township health centers and community health service centers. It can be seen that the current reform of the medical community has not yet played a big role in the allocation of medical infrastructure, especially the resources of rural medical facilities. Grassroots medical infrastructure is also an important factor to attract talents and superior medical personnel to the grass-roots level. Therefore, the allocation of primary medical resources not only requires the overall grasp of the medical community, but also requires the financial departments and health departments to improve the grassroots health infrastructure and resource allocation subsidies.

6.2.3.Enhancing Incentive Measures for Health Personnel in Terms of Salary and Staffing

Based on the analysis in this paper, the construction of the county medical community in Sichuan Province has not effectively influenced the allocation of medical human resources. One possible explanation for this is that the medical community has not made substantial changes to the establishment of positions to encourage the flow of higher-level health personnel, thereby failing to significantly impact the allocation of healthcare human resources. To address this, it is essential to further refine the salary incentive mechanism and staffing arrangements for healthcare professionals. These adjustments will be instrumental in attracting medical talent to participate in the development of county-level compact medical communities, especially in building the capacity of primary healthcare services.

References:

[1]Wei YX,Wang ZY.Effect analysis and thinking of close medical joint trusteeship cooperation in Zhejiang Province--Based on incomplete contract theory[J].Health Economics Research,20,37(09):16-19.

[2]Huang Y,Zhang L Y.Incentive Compatibility:The realization path of"Graded Diagnosis and Treatment"in China--a case study based on the reform of the Medical community in S County[J].Chinese Journal of Administration,2019,No.409(07):115-123.

[3]Wang HB.Reform Model,Challenges and Countermeasures of county and Township

medical service system integration[J].Chinese Health Economics,2017,36(12):78-81.
[4] Fan SG, Wei L, Yu CY, etc. Study on the difference in integration ability and stability of county medical association under different cooperative relations [J]. Health Economic Research, 2020,37 (04): 25-29.
[5]The Development Research Center of the State Council Experience,Problems and Suggestions for promoting Graded diagnosis and treatment[M].Beijing:China Development Press,2017:11-12.
[6]Jonas S.An Introduction to the US Health Care System[M].New York:Springer Pub Co,2003:102-104.
[7]Shen SG,Du L.What kind of hierarchical diagnosis and treatment do we need[J]?Social Security Review,2019.3(04):70-82.
[8]Shen SG,Zhang B.Hierarchical diagnosis and treatment,primary diagnosis and construction of primary medical and health institutions[J].Xuehai,2016(2):48-57.
[9]GAO HR.Health management and hierarchical diagnosis and treatment system in China[J].Journal of Public Administration,2017,14(02):139-144+159.(in Chinese)
[10]Zhang HL,Cheng CH,Ma XE.Status analysis and countermeasures of hierarchical diagnosis and treatment system[J].China Hospital Management,2015,35(11):8-9.
[11]Du C,Zhu HP.Evolution logic of urban health care system in China.Social Sciences in China,2016(08):66-89+205-206.
[12]Wang M,Yu GL,Huang YM et al.Research on implementation obstacles and coping strategies of hierarchical diagnosis and treatment system[J].Health Economics Research,2017(04):15-18.
[13] Zhu HP. The root cause of the difficulty of hierarchical diagnosis and treatment is that medical treatment is not in medical insurance [J]. Medical Insurance of China, 2017 (05): 20-22.
[14]Stephen M..Shortell,Robin R.Gillies,and David A.Anderson.Remaking Healthcare in America:The Evolution of Organized Delivery Systems[M].San Francisco:Jossey-Bass,2000:56-59.
[15]Fisher E.S.,Shortell S.M.,Kreindler S.A.,et al.A Framework for Evaluating the Formation,Implementation,And Performance of Accountable Care Organization[J].Health Affairs,2012,31(11):2368-2378.
[16]Nyweide D.J.,Lee W.,Cuerdon T.T.,et al,Conway PH.Association of Pioneer Accountable Care Organizations vs traditional Medicare fee for service with spending,utilization,and patient experience[J].JAMA.2015,313(21):2152-2161.
[17]Gu X,Guo K.From pay-per-item to pay-per-value:Reform of Medicare Payment System in the United States[J].Journal of Dongyue,2018,39(10):79-87.
[18]Li L,Xu Y,Chen Q.Integrated medical care:a strategic choice of medical reform in China[J].China Health Policy Research,2012,5(09):10-16.
[19]Jiang XM,Liang RH.[J]Research on countermeasures to improve the construction of medical Union in China--based on transaction cost theory[J].Xinjiang Social Sciences,2019(06):141-146.
[20]Xiong W,Gao C,Zhang Y et al.Development strategy of large public hospitals under the background of hierarchical diagnosis and treatment[J].Soft Science of Health,2018,32(08):37-39.(in Chinese)

[21]Liang WN,Wang C,Wu PX.Chinese Academy of Medical Sciences.Blue Book of Medical Reform:China Medical Reform Development Report(2020)[R].Beijing:Chinese Academy of Medical Sciences and Social Sciences Academic Press,2020:123-137.

[22]Li L,Chen JF.Review on the progress of the new medical reform:An analysis based on historical perspective and global perspective[J].Social Security Research,2012,15(01):107-115.

[23]Curry N.,Ham C..Clinical and Service Integration:the Route to Improved Outcomes[R].London:(Retrieved from)The Kings Fund,2010:23-26.

[24]Zhu JM,Duan H.How can county medical communities achieve health performance?--Policy entrepreneurs,reorganized alliances and incentive compatibility[J].Journal of Public Administration,201,18(03):125-138+174-175.(in Chinese)

[25]Wang HX,Chen ZY.Wang HX,Chen ZY.The influence of medical consortium construction on the effect of graded diagnosis and treatment in China[J].Journal of Nanjing Medical University(Social Sciences Edition)2019,19(2):90-93.(in Chinese)

[26]Wang XF,Liu WY,Niu CL et al.Evaluation and analysis of operation effect of medical union:A case study of a district in Beijing[J].Chinese Journal of Medical Sciences,2019,10(05):225-229.

[27]Feng Jin,Lv SNUr,Wang Zhen.Medical resource sharing and patients'choice of medical treatment:Policy evaluation of medical consortium construction in China[J].Management World,2012,38(10):144-157+173+158.

[28]Liang WN,Wang C,Wu PX.Chinese Academy of Medical Sciences.Blue Book of Medical Reform:China Medical Reform Development Report(2020)[R].Beijing:Chinese Academy of Medical Sciences and Social Sciences Academic Press,2020:325-340.

[29]Wang SY,Sun ZS.Effect of hierarchical diagnosis and treatment reform on patient satisfaction and diagnosis and treatment choice behavior.Journal of Management Engineering,2021,35(6):115-127.

[30]Shen LJ,Huang CF,Li LL et al.Exploration and practice of county medical community model:A case study of Tianchang City,Anhui Province[J].Health Economics Research,2018,No.380(12):7-11.

[31]Liu Y,Xu P,Wang XL.Practice and effect of county medical community construction in agricultural and pastoral areas of northern Tibet:an empirical study[J].Chinese Tibetology,2022,150(01):106-115+217.

[32]Zhu J M,Duan H.How can county medical communities achieve health performance?--Policy entrepreneurs,reorganized alliances and incentive compatibility[J].Journal of Public Administration,201,18(03):125-138+174-175.(in Chinese)

[33]Liao DP,Lan Z,Liu J et al.Study on the impact of medical insurance package payment on the operation and management of compact medical community[J].China Health Economics,2023,42(05):29-33.

[34]Bai R,Lv Y.Research on implementation obstruction of medical Union policy from the perspective of policy network[J].Health Economics Research,2019,36(12):11-13.

[35]Feng QL.Human Health Community:Value-oriented development of the Yangtze River Delta regional medical Union[J].Social Sciences of Nanjing,2021,No.403(05):63-70.

[36]Huang XG,Wang LZ,Yu GL et al.Study on the model of medical association and its development[J].Health Economics Research,2016(03):10-12.

[37]Zhang RH,Zhao DR,He SC et al.Analysis and reflection on the practice of medical commonwealth in China[J].Health Economics Research,2016(06):12-15.
[38]Duan H,Zhang YN,Hou Yucheng et al.[J]Study on the influence of Chinese medical Commonwealth policy on the service ability of grassroots community medical and health institutions:a quasi-experimental study from the perspective of organizational alliance theory.Journal of Gansu Administration College,2020(04):4-16+124.
[39]Jiang XM.Ling RH.Research on countermeasures to improve the construction of medical Consortium in China--Based on the perspective of transaction cost.Social Sciences,2019,223(6):141-146.
[40]Xu YY,Yu JX.The reform of medical insurance payment and the implementation of Strong grass-roots Strategy:the experience of Zhejiang County Medical Community[J].Chinese Administration,2020,No.418(04):102-108.
[41]Wu YX,Li YP.Effect of package payment on patient flow of medical care in compact county medical community.Journal of Nanjing Medical University(Social Sciences Edition),2023,23(02):144-149.
[42]Li L,Zheng Y.Analysis of incentive and constraint mechanism of county medical community under total prepayment system:Conflict between individual interests and collective interests[J].Review of Public Administration and Policy,2019,12(01):140-155.
[43]Sun Y,Gu XF,Feng YM.Research on Coordination mechanism between medical insurance payment method and salary system reform of public hospitals--An Analysis of salaried doctors'behavior based on Expectation Theory[J].China Health Policy Research,2018,11(12):45-50.
[44]Hurwicz L..The Design of Mechanisms for Resource Allocation[J].The American Economic Review,1973(63):1-30.
[45][E]Morris.Selected Papers of James Morris(Motivation Theory under Asymmetric Information)[M].Trans.Zhang Weiying.Beijing:The Commercial Press,1997.
[46]Hu HW,Liu GE.The effect and mechanism of urban residents'medical insurance on national health.Southern Economy,2012(10):186-199.
[47]Gao QM,Wang TY.Can differentiated reimbursement ratio design promote graded diagnosis and treatment?--Evidence from inpatient reimbursement data[J].Insurance Research,2018(7):89-103.
[48]Zhou HQ,Li AF,Du YQ,et al.Zhou Hai-Q,LiAi-Fang,et al.Effect evaluation of trustee-type intensive medical union on promoting hierarchical diagnosis and treatment[J].Chinese Journal of Continuing Medical Education,2018,10(28):52-55.
[49]Fang SH.Research on the path to realize hierarchical diagnosis and treatment under the background of universal Medical insurance[C].Annual Conference of China Health Economics Society.2013.
[50]Peter D.,Vikram P..Can increased primary care access reduce demand for emergency care?Evidence from England's 7-day GP opening[J].Journal of Health Economics,2016(49):193-208.
[51]Shi DQ,Ding H,Wei P et al.Whether smart city construction can reduce environmental pollution.China Industrial Economics,2018(06):117-135.]
[52][Sun LL,Yang H,Zheng HT.The impact of land ownership confirmation on Chinese farmers'capital investment:a micro-analysis based on the heterogeneity of

farmers'model[J].Economic Research Journal,2019,55(11):156-173.
[53]Jcobsona L.S.,LaLonde R.J.,Earnings Losses of Displaced Workers[J].American Economic Review,1993,83(4):685-709.
[54]Liu RM,Zhao RJ.Do national high-tech zones promote regional economic development?--Verification based on difference-difference method[J].Management World,2015(08):30-38.
[55]Cai,X.,Lu Y.,Wu M.,et al.Does Environmental Regulation Drive away Inbound Foreign Direct Investment?Evidence from a Quasi-Natural Experiment in China[J].Journal of Development Economics,2016(123):73-85.

Supporting information

S1 FIG.(png)

S2 File.Placebo test.(docx)

S3 File. Data sample.(xlsx)

Click here to access/download
Supporting Information
S1 FIG 1.png

Click here to access/download
Supporting Information
S2 File.Placebo test.docx

Click here to access/download

Supporting Information

S3 File. Data sample.xlsx
