# Supplementary material for: County medical community, medical insurance package payment, and hierarchical diagnosis and treatment—Empirical analysis of the impact of the pilot project of compact county medical communities in Sichuan Province
Source: PLoS One. 2024 Apr 5;19(4):e0297340. doi: 10.1371/journal.pone.0297340 (PMC10997099; doi:10.1371/journal.pone.0297340)
Supplement: Attachment — Submitted filename: Response to Reviewer2.docx [file pone.0297340.s006.docx]

**Subject: Response to Reviewer #2's Comments on Manuscript PONE-D-23-37259**

Dear Prof. Akande,

Thank you for sharing the insightful comments regarding our manuscript titled "County Medical Community, Medical Insurance Package Payment, and Hierarchical Diagnosis and Treatment" with the ID PONE-D-23-37259. We appreciate the positive feedback on several sections of our paper and have taken great care to address the concerns raised. Below, I provide a detailed response to each of the your comments:

**1.Comment on Abstract's Conclusion:**

**Reviewer's Comment:** "Well written but need to include key policy implication in the concluding part of the abstract."

**Response:** We agree with the reviewer that the policy implications are crucial in the abstract. We have revised the abstract to include a concise statement on the key policy implications derived from our study. This revision can be found at the end of the abstract in page 2.

**2.Comment on the Lack of Discussion:**

**Reviewer's Comment:** "There is no discussion on this manuscript. Findings / Result of this study were not discussed at all. The findings were not explained and not compared with findings from other studies."

**Response:** We acknowledge the reviewer’s concern regarding the absence of a discussion section. To rectify this, we have added a comprehensive discussion section where we interpret our findings, compare them with existing literature, and explore their broader implications. This new section can be found in page 23-24.

**3.Comment on Conclusion and Policy Implications:**

**Reviewer's Comment:** "Clearly written but without very clear policy implication and recommendation."

**Response:** We appreciate the reviewer's emphasis on the importance of policy implications and recommendations in the conclusion. Accordingly, we have revised the conclusion to more explicitly state the policy implications and recommendations stemming from our research. These additions are now clearly articulated in page 25, the concluding section.

We hope that these revisions adequately response your concerns and enhance the overall quality and impact of our manuscript. We are grateful for the opportunity to improve our work and thank you for the constructive feedback.

We look forward to the possibility of our manuscript being accepted for publication in PLOS ONE and believe it will make a meaningful contribution to the field.

Thank you for your time and consideration.

Happy New Year 2024 in Advance!

Sincerely,

Yuxuan Zhou

School of Public Administration, Southwestern University of Finance and Economics, Chengdu, Sichuan, China

Liulin Campus, Southwestern University of Finance and Economics, 555 Liutai Avenue, Wenjiang District, Chengdu , Sichuan, China

E-mail: 15556932867@163.com (Zhou)

On December 26, 2023
